# Supplementary material for: Combining seasonal malaria chemoprevention with novel therapeutics for malaria prevention: a mathematical modelling study
Source: PLoS Comput Biol. 2026 Feb 26;22(2):e1014021. doi: 10.1371/journal.pcbi.1014021 (PMC12962535; doi:10.1371/journal.pcbi.1014021)
Supplement: S1 Text — (PDF) [file pcbi.1014021.s001.pdf]

Supplement to: Combining seasonal malaria chemoprevention with novel therapeutics for malaria prevention: a mathematical modelling study

Lydia Braunack-Mayer[1, 2], Josephine Malinga[1,3], Narimane Nekkab[4], Sherrie L Kelly[4], Jörg J Möhrle[4, 5], Melissa A Penny[1, 3, *]

1 The Kids Research Institute Australia, Nedlands, WA, Australia

2 Institute of Social and Preventive Medicine, University of Bern, Bern, Switzerland

3 Centre for Child Health Research, The University of Western Australia, Crawley, WA, Australia

4 Swiss Tropical and Public Health Institute, Allschwil, Switzerland

5 University of Basel, Basel, Switzerland

* Correspondence to:

Prof Melissa A Penny

melissa.penny@uwa.edu.au

Contents

1. Methods 3

2. Additional results: Combining SMC with seasonal deployment of a pre-liver stage product 10

3. Additional results: Combining SMC with seasonal deployment of a blood stage product 20

4. Additional results: Combining SMC with seasonal deployment of a multi-stage product 23

5. References 28

1. Methods

Malaria transmission model

We used an established individual-based model of malaria transmission, OpenMalaria, to evaluate the benefits of combining seasonal deployment of a novel medical prevention intervention with seasonal malaria chemoprevention (SMC). OpenMalaria was originally developed to estimate the potential public-health impact of a malaria vaccine [1]. This stochastic model is open source (https://github.com/SwissTPH/openmalaria/wiki) and has been described previously [1,2]. Our study used OpenMalaria version 45 (https://swisstph.github.io/openmalaria/schema-45.html). In Table A, we provide a summary of the key model dynamics relevant for this study.

**Name**	**Description and assumptions**	**References**
**Key modelled epidemiological processes**		
Human malaria infection	• The force of infection in the simulated setting is determined by an inputted entomological inoculation rate (EIR)<br>• EIR inputs were defined as 1, 4, 8, 16, and 32 for this study<br>• Human exposure to mosquitoes depends on age	[1,3]
Infection progression in humans, including asexual parasite densities and immunity	• OpenMalaria includes several within-host models of asexual parasite densities<br>• This study made use of an empirical model where expected densities of a single infection are sampled from a log-normal distribution and calibrated to malaria therapy data<br>• The model captures between and within-host variation and the duration of infection follows a log-normal distribution<br>• Blood stage parasite density depends on the time since infection and is influenced by naturally acquired immunity<br>• Both pre-erythrocytic and blood stage immunity develop following consequent episodes of exposure to infection and decay exponentially<br>• Acquired immunity acts to reduce the parasite density of subsequent infections<br>• Multiple infections are possible with cumulative parasite densities	[1,3,4]
Transmission from infected humans to mosquitoes	• Transmission depends on the density of parasites present in the human, with gametocyte densities following between ten and 20 days after asexual infection	[1,5,6]

Name	Description and assumptions	References
Clinical illness, morbidity, mortality, and anaemia	● Acute clinical illness depends on the human host's parasite densities and their pyrogenic threshold, which evolves over time depending on the individual's history of exposure<br>● Acute episodes of morbidity can be uncomplicated or can evolve to severe episodes<br>● In this study, uncomplicated malaria was defined as an episode of symptomatic malaria, detectable by rapid diagnostic test with 94.2% specificity and a detection limit of 50 parasites per microlitre, where symptoms did not qualify as severe malaria<br>● An episode of severe malaria was defined as an episode of symptomatic malaria, detectable by rapid diagnostic test and with symptoms qualifying as severe malaria or with age-related comorbidities<br>● A proportion of severe episodes lead to death<br>● Prevalence was calculated according to the number of patent infections detectable by rapid diagnostic test with 94.2% specificity and a detection limit of 50 parasites per microlitre	[1,7–9]
**Modelled characteristics of the transmission setting**		
Population	● 10 000 individuals were monitored for this study<br>● The population's age structure for this study was informed by health and demographic surveillance data from Tanzania	[9,10]
Sex and gender	● Model dynamics and outputs are not disaggregated by sex or gender, due to the lack of appropriate data for model calibration to these characteristics	
Transmission seasonality	● Transmission is seasonally forced, reproducing the same seasonal pattern each year in absence of interventions<br>● Two seasonal settings were used for this study: fourier series where 70% of cases occurred within four and six months of the year, respectively	[3,11]
Case management	● Case management is modelled through a comprehensive decision tree-based model, which determines treatment implications depending on the occurrence of clinical events, such as fever and care seeking<br>● The case management model includes the specification of diagnostic tests, the effects of treatment, case fatality, case sequelae, and cure rates<br>● In this study, cases were determined according to rapid diagnostic test with a parasite detection limit of 50 and 94.2% specificity	[12]
Entomological setting	● Comprehensive simulation of the mosquito life cycle and behavior towards human and animal hosts (including biting and resting) is embedded in a dynamic entomological model of the mosquito oviposition cycle	[13]

Name	Description and assumptions	References
	● Multiple vector species can be simulated simultaneously<br>● This study used archetypal dynamics for Anopheles gambiae with indoor and outdoor biting behaviour	
**Modelled interventions**		
Vector control	● Available interventions are: long-lasting insecticide-treated nets (LLINs), indoor residual spraying (IRS), house screening, baited traps, repellents, and push-pull	[13]
Drugs and Vaccines	● Drug- and vaccine-based interventions can be deployed at various levels of the parasite life cycle (transmission blocking, anti-infective, blood stage clearance)	[14]
Deployment characteristics	● In addition to case-management of clinical cases, interventions can be deployed in several ways:<br>○ Continuously to individuals by age, for example to infants for an expanded program of immunisation or for perennial malaria chemoprevention<br>○ At specified times in a year or over multiple years, to targeted group of individuals (for example by age) for several cycles and specified coverages<br>● Interventions can also be deployed by enrolling individuals into cohorts and tracking cohort outcomes, facilitating clinical trial simulation	a
**Simulation regimes and model variants**		
Time steps	● Simulation outputs are tracked every five days	
Model variants	● Model assumptions regarding immunity decay, treatment, and heterogeneity of transmission can be varied, resulting in 14 model variants<br>● For this study, we used OpenMalaria's base model variant	[15]
**Software availability and documentation**		
[a]Source code and wiki page available on GitHub: https://github.com/SwissTPH/openmalaria/		

Table A: Summary of disease model characteristics, adapted from earlier OpenMalaria publications [2,16,17]

Outcome measures

To evaluate the benefits of adding a new therapeutic to an existing malaria prevention program, we measured reductions in the cumulative incidence of uncomplicated and severe malaria throughout childhood. The calculations for these metrics involved the following steps:

1. Using OpenMalaria, we simulated disease outcomes for a cohort of children born in the first year of intervention delivery for a ten-year period.
2. We evaluated the cumulative incidence of uncomplicated and severe malaria cases when children reached five and ten years old.
3. As illustrated in Fig A, we captured these cumulative incidences under three scenarios: children received only SMC, children received only the novel therapeutic, and children received both SMC and the novel therapeutic.
4. The protective efficacy of the combined intervention was then calculated relative to two counterfactual scenarios, as

$$\mathrm{CI}_{\mathrm{SMC}} - \mathrm{CI}_{\mathrm{combination}} \,/\, \mathrm{CI}_{\mathrm{SMC}}$$

or as

$$\mathrm{CI}_{\mathrm{novel\ therapeutic}} - \mathrm{CI}_{\mathrm{combination}} \,/\, \mathrm{CI}_{\mathrm{novel\ therapeutic}}$$

for $\mathrm{CI}_{\mathrm{SMC}}$ the cumulative incidence of uncomplicated or severe malaria cases by five or ten years old for children receiving only SMC, $\mathrm{CI}_{\mathrm{novel\ therapeutic}}$ the cumulative incidence for children receiving only the novel therapeutic, and $\mathrm{CI}_{\mathrm{combination}}$ the cumulative incidence for children receiving the combined intervention.

These relative measures of impact were chosen because they capture the likely benefits of a seasonally deployed intervention as it would be measured in phase three or four clinical trials. For example, the R21/Matrix-M phase three clinical trial evaluated vaccine efficacy relative to a control arm where children received regular deployment of SMC [18]. The phase three trial of seasonal vaccination with RTS,S/AS01E with or without SMC also reported the protective efficacy of the combined interventions relative to both children who received only RTS,S/AS01E and only SMC [19].

Intervention dynamics and deployment

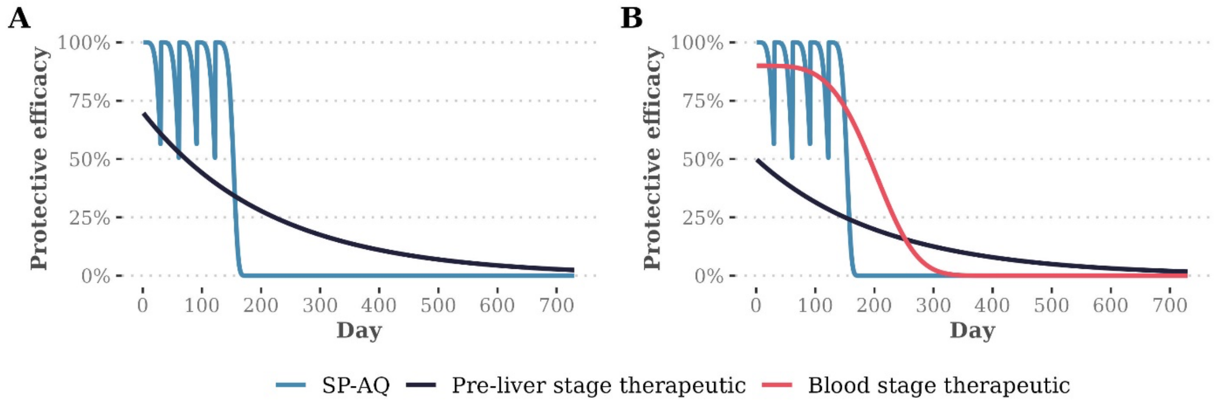

Fig A. Illustration of key model parameters

Key model parameters included the component’s initial efficacy at the time of administration, the protection half-life or number of days until therapeutic efficacy reached half of its initial efficacy, and the shape of efficacy decay. Initial efficacy refers to the therapeutic’s effect at time of administration as a percentage of the maximum possible effect. Protection half-life is the number of days until the effect decays to 50% of its initial efficacy. This parameter value is the scale parameter used in the Weibull function for effect decay. The decay shape is the shape parameter used in the Weibull function for effect decay. Panel A illustrates five cycles of SMC with SP-AQ together with a pre-liver stage therapeutic with 70% initial efficacy, a protection half-life of 150 days, and a long tail of protection (decay shape parameter of 1). Panel B illustrates five cycles of SMC with SP-AQ together with a multi-stage therapeutic with: 70% pre-liver stage initial efficacy, a pre-liver stage protection half-life of 150 days, a long pre-liver stage tail of protection (decay shape parameter of 1), 90% blood stage initial efficacy, a blood stage protection half-life of 200 days, and a rapid blood stage tail of protection (decay shape parameter of 4).

Statistical analysis

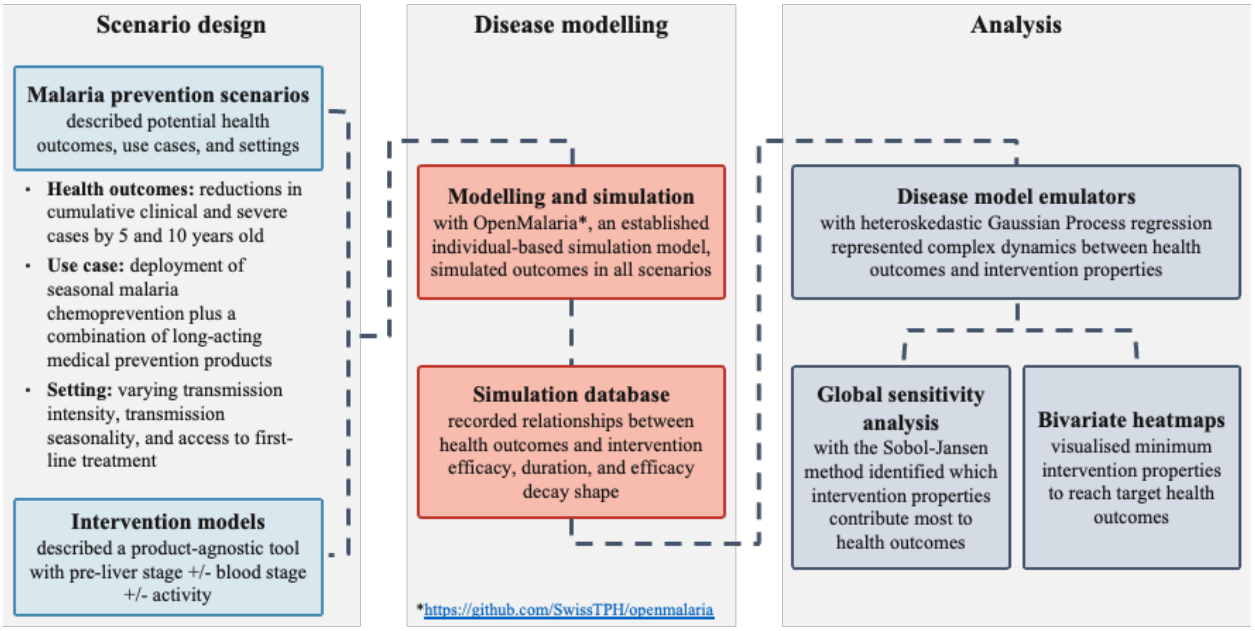

Fig B: Schematic diagram of analysis framework for defining essential product characteristics for malaria prevention interventions

Following our previously published methodology [16], and as illustrated in Fig B, we applied a predictive target product profile modelling framework to identify the therapeutic properties associated with maximum public health benefit when combining SMC with novel interventions.

We began by generating simulations from the OpenMalaria individual-based transmission model across a discrete sample of input intervention parameters. A total of 500 parameter sets were generated using Latin hypercube sampling with a uniform probability distribution, ensuring uniform coverage of a multidimensional input space. Parameter sets were sampled from the ranges shown in table 1, which were chosen to represent the broadest possible space of likely properties of a next-generation prevention therapeutic. For each sampled intervention profile, defined by the values for initial efficacy, protection half-life, and decay shape, we simulated ten stochastic replicates to capture stochastic variation in model outcomes.

Next, we trained a heteroskedastic Gaussian Process regression model using the hetGP package in R [20] to emulate the relationship between input intervention parameters and simulated health outcomes (i.e., cumulative incidence of uncomplicated and severe malaria). This surrogate model, known as a model emulator, approximates the complex and computationally intensive dynamics of the individual-based model without needing to perform a full simulation,

allowing computationally efficient evaluation across a broader parameter space. Emulators were trained separately for each model scenario (type of therapeutic, seasonality profile, level of access to care and deployment scenario).

Gaussian Process regression models have previously been used successfully to emulate therapeutic dynamics in OpenMalaria in this exact framework [16,17,21]. Accordingly, we did not perform additional cross-validation for model selection. Instead, we assessed emulator performance using the R-squared correlation coefficient between observed and predicted values on a 10% hold-out validation set. To further confirm model fit, we examined both the R-squared values and scatter plots of true versus predicted outcomes.

To assess the influence of each intervention property on model outcomes, we conducted a nonparametric variance-based sensitivity analysis using the sensitivity package in R [22]. We applied the Sobol-Jansen method [23] to estimate Sobol total-order indices, which quantify the contribution of each input parameter, including all interaction effects, to the variance of the output. This analysis was performed on two large input datasets (50,000 parameter samples each) generated via Latin hypercube sampling, with 1000 bootstrap replicates used to estimate uncertainty.

Finally, to identify minimum required therapeutic properties for achieving target health outcomes, we used the trained emulators to create bivariate heatmaps. For each model scenario, emulators were used to predict health outcomes for a grid of therapeutic properties (e.g., protection half-life, initial efficacy). The predictions were used to generate heatmaps, allowing us to identify the minimum value of each property (e.g., protection half-life) for which the mean prediction remained above a chosen public health target (e.g., ≥10% reduction in cumulative severe malaria by age 10).

All statistical analyses were conducted in R (version 4.3.2) [24]. Calculations were performed at sciCORE (http://scicore.unibas.ch/) scientific computing centre at the University of Basel.

2. Additional results: Combining SMC with seasonal deployment of a pre-liver stage product

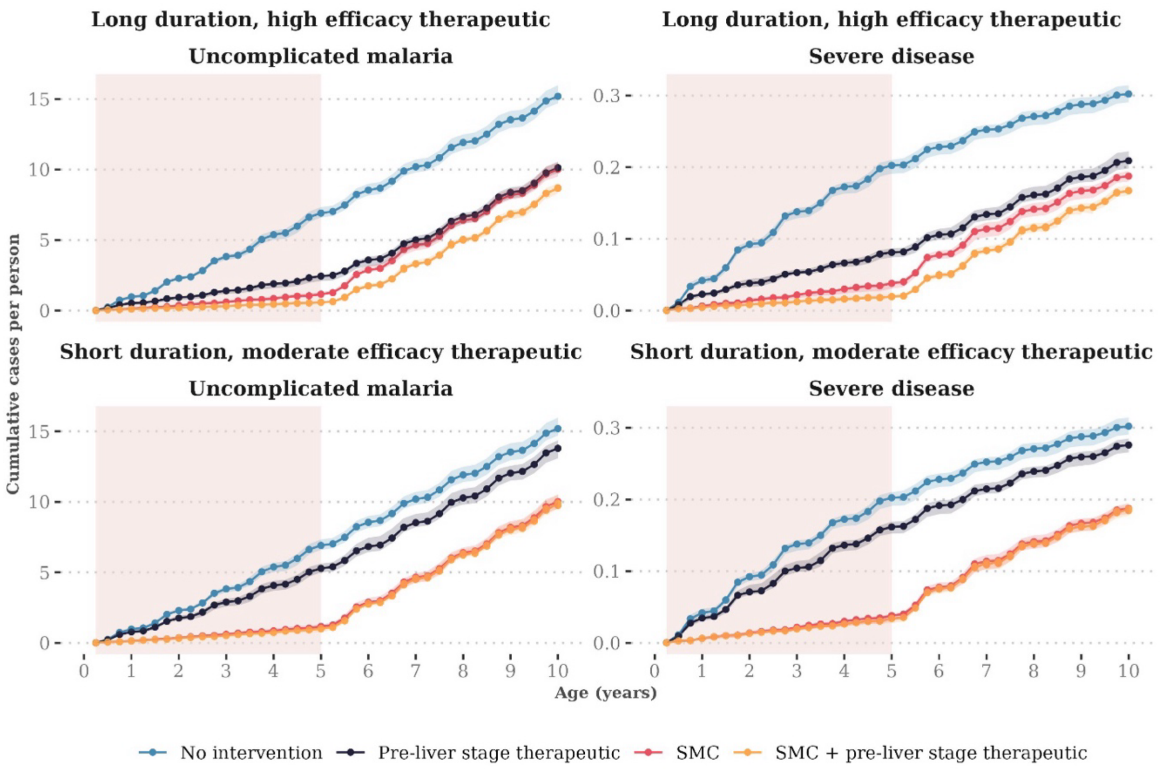

Fig C: Predicted cumulative case curves when seasonal deployment of two exemplar pre-liver stage products are combined with perfect deployment of SMC

The median number of cumulative cases per year of age were calculated when children aged three to 59 months received one of the following interventions: no intervention, five SMC cycles (perfect deployment scenario), seasonal deployment of a pre-liver stage therapeutic, or the combination of five SMC cycles and the pre-liver stage therapeutic. Top panels include deployment of a pre-liver stage therapeutic with a protection half-life of 354 days, 90% initial efficacy, and decay shape parameter of 1. Bottom panels include a pre-liver stage therapeutic with a protection half-life of 120 days, 50% initial efficacy, and decay shape parameter of 1. The pink shaded regions indicate age-eligibility for SMC and the novel therapeutic. Shaded regions indicate the minimum and maximum cumulative cases per person observed across ten stochastic replicates of simulations from the individual-based malaria transmission model. Model results are shown for a scenario with transmission intensity corresponding to 32% *Pf*$PR_{2\text{-}10}$, where 75% of malaria cases occur within six months of the year and the probability of seeking first-line treatment for clinical malaria over 14 days is low (10%).

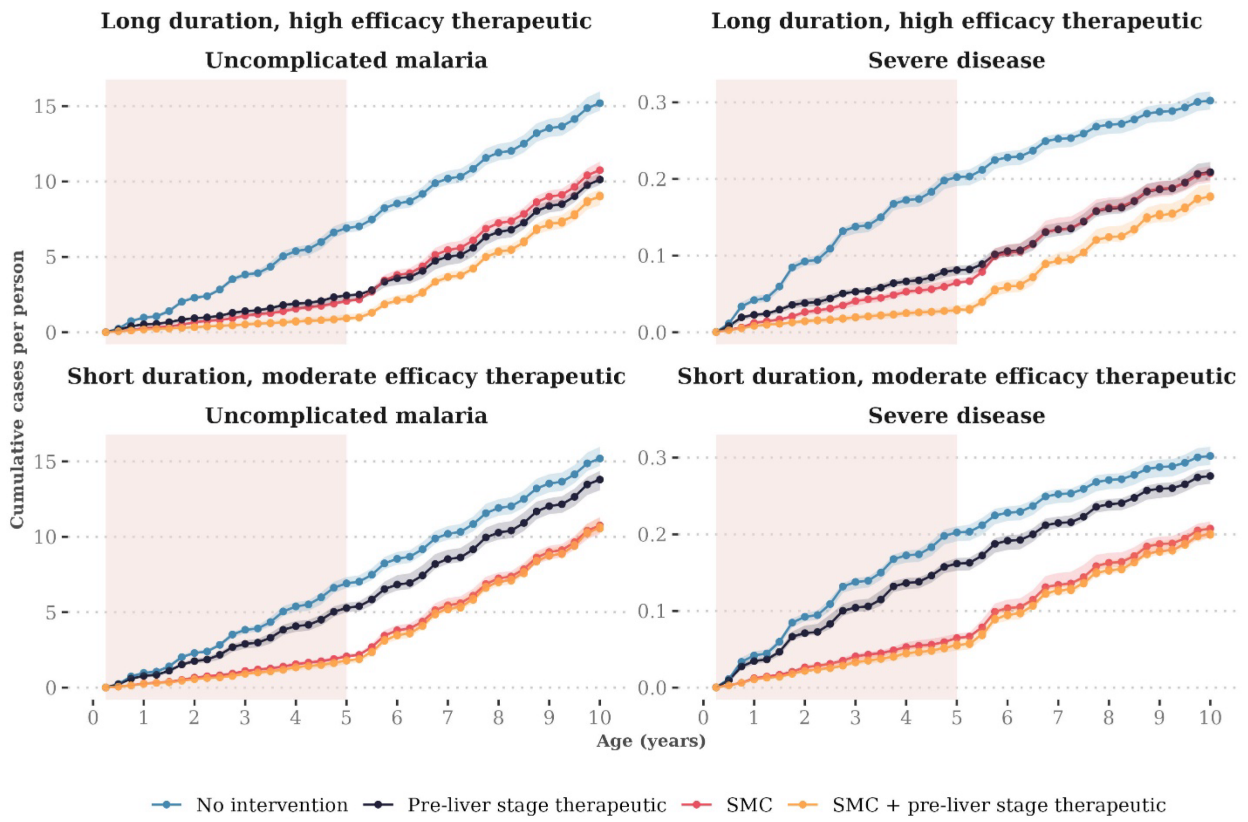

Fig D: Predicted cumulative case curves when seasonal deployment of two exemplar pre-liver stage products are combined with imperfect seasonal coverage of SMC

The median number of cumulative cases per year of age were calculated when children aged three to 59 months received one of the following interventions: no intervention, four SMC cycles (imperfect seasonal coverage scenario), seasonal deployment of a pre-liver stage therapeutic, or the combination of four SMC cycles and the pre-liver stage therapeutic. Top panels include deployment of a pre-liver stage therapeutic with a protection half-life of 354 days, 90% initial efficacy, and decay shape parameter of 1. Bottom panels include a pre-liver stage therapeutic with a protection half-life of 120 days, 50% initial efficacy, and decay shape parameter of 1. The pink shaded regions indicate age-eligibility for SMC and the novel therapeutic. Shaded regions indicate the minimum and maximum cumulative cases per person observed across ten stochastic replicates of simulations from the individual-based malaria transmission model. Model results are shown for a scenario with transmission intensity corresponding to 32% *Pf*PR$_{2\text{-}10}$, where 75% of malaria cases occur within six months of the year and the probability of seeking first-line treatment for clinical malaria over 14 days is low (10%).

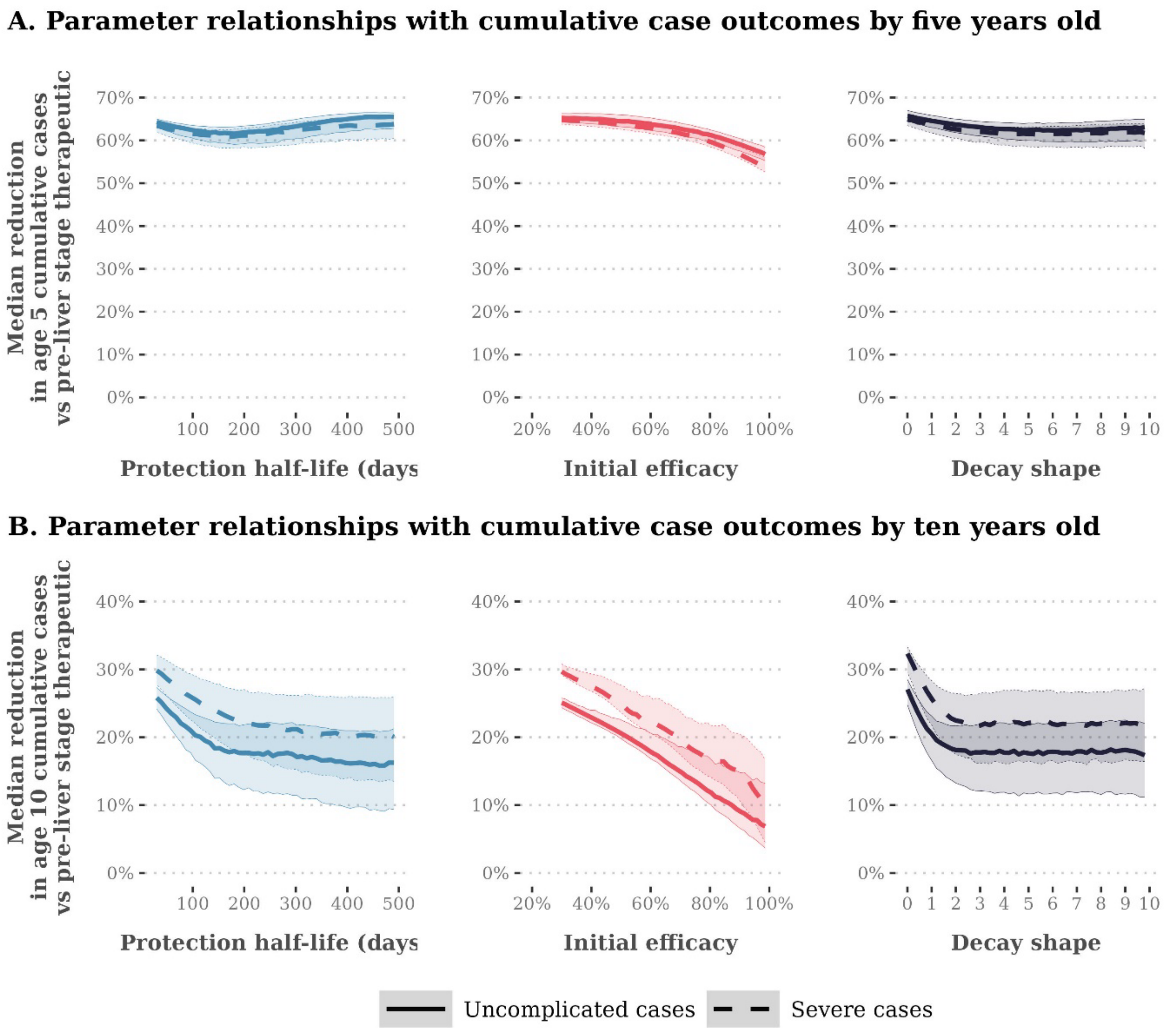

Fig E: Gaussian Process regression emulator predictions for the relationship between pre-liver stage therapeutic properties and expected reductions in cumulative uncomplicated and severe cases when deployed with SMC, evaluated relative to seasonal deployment of a pre-liver stage therapeutic alone

Results show the imperfect deployment scenario, where children received three SMC cycles. Transmission is high (32% *Pf*$PR_{2\text{-}10}$), 75% of malaria cases occur within six months of the year, and the probability of seeking first-line treatment for clinical malaria over 14 days is low (10%). Each panel shows the median reduction in cumulative uncomplicated (solid lines) or severe cases (dashed lines) achieved by combining seasonal deployment of a pre-liver stage therapeutic with SMC, relative to cumulative cases when the pre-liver stage therapeutic is deployed alone. Median reductions are calculated by dividing the parameter range for the therapeutic property shown on the x-axis

(protection half-life, initial efficacy, decay shape) into 51 segments, and calculating the median outcome for each given segment across all other parameter values. Shaded regions represent the 25% and 75% quantiles of the corresponding reductions. Emulator predictions are shown separately for cumulative case outcomes at five (panels A) and ten years old (panels B). For outcomes at five, reductions of approximately 65% were observed across the full range of pre-liver stage parameter values, which is similar to the protective efficacy observed for the combination of RTS,S and SMC relative to SMC alone (59·0%, 95% CI 54·7 to 62·8).[19] This near-constant additional benefit is likely because SMC provides a curative antimalarial drug and is thus able to clear infections that escape the pre-liver stage product. The large size of the benefit is because we have evaluated SMC’s impact relative to the benefit of the pre-liver stage therapeutic deployed alone; in absolute terms, the additional number of cases averted by SMC will decrease with increasing efficacy and duration of the novel therapeutic.

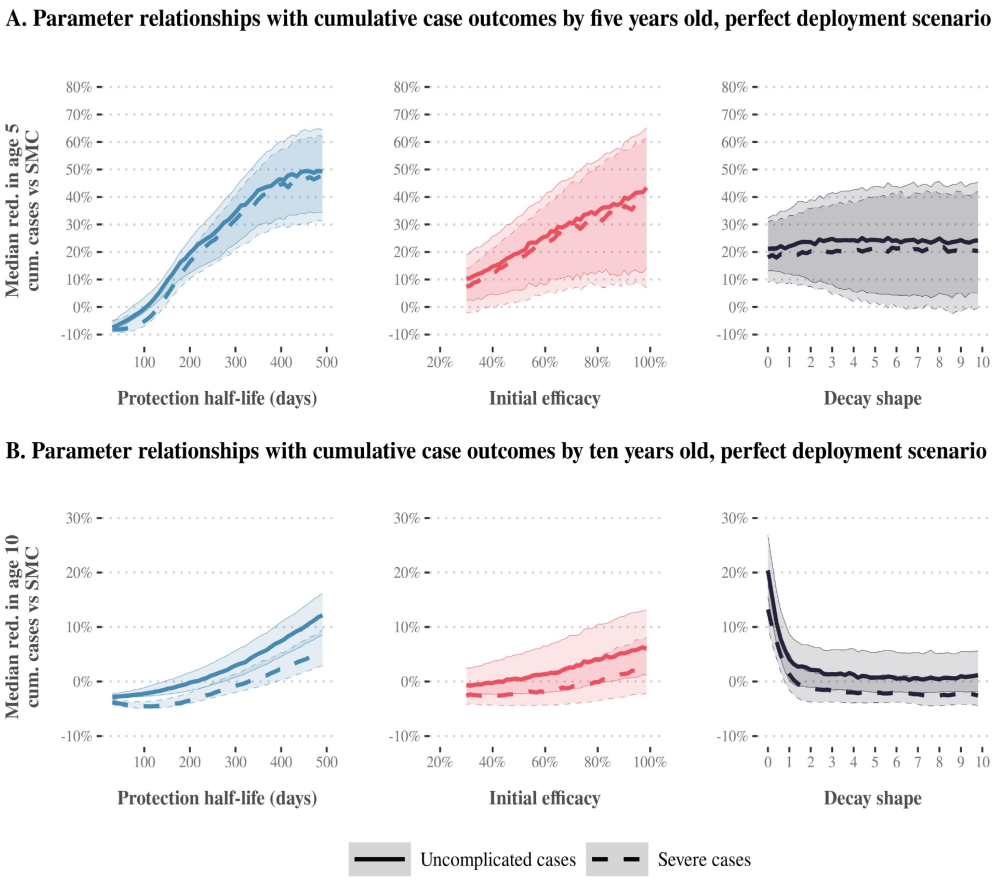

Fig F: Gaussian Process regression emulator predictions for the relationship between pre-liver stage product properties and expected reductions in cumulative uncomplicated and severe cases relative to SMC alone, evaluated with perfect coverage of SMC

Results show the perfect deployment scenario, where children aged three to 59 months received five SMC cycles. Transmission is high (32% *Pf*PR$_{2-10}$), 75% of malaria cases occur within six months of the year, and the probability of seeking first-line treatment for clinical malaria over 14 days is low (10%). Each panel shows the median reduction in cumulative uncomplicated (solid lines) or severe cases (dashed lines) achieved by combining seasonal deployment of a pre-liver stage product with SMC, relative to cumulative cases when SMC is deployed alone. Median reductions are calculated by dividing the parameter range for the product property shown on the x-axis (protection half-life, initial efficacy, decay shape) into 51 segments, and calculating the median outcome for each given segment across all other parameter values. Shaded regions represent the 25% and 75% quantiles of the corresponding reductions. Emulator predictions are shown separately for cumulative case outcomes at five (panels A) and ten years old (panels B).

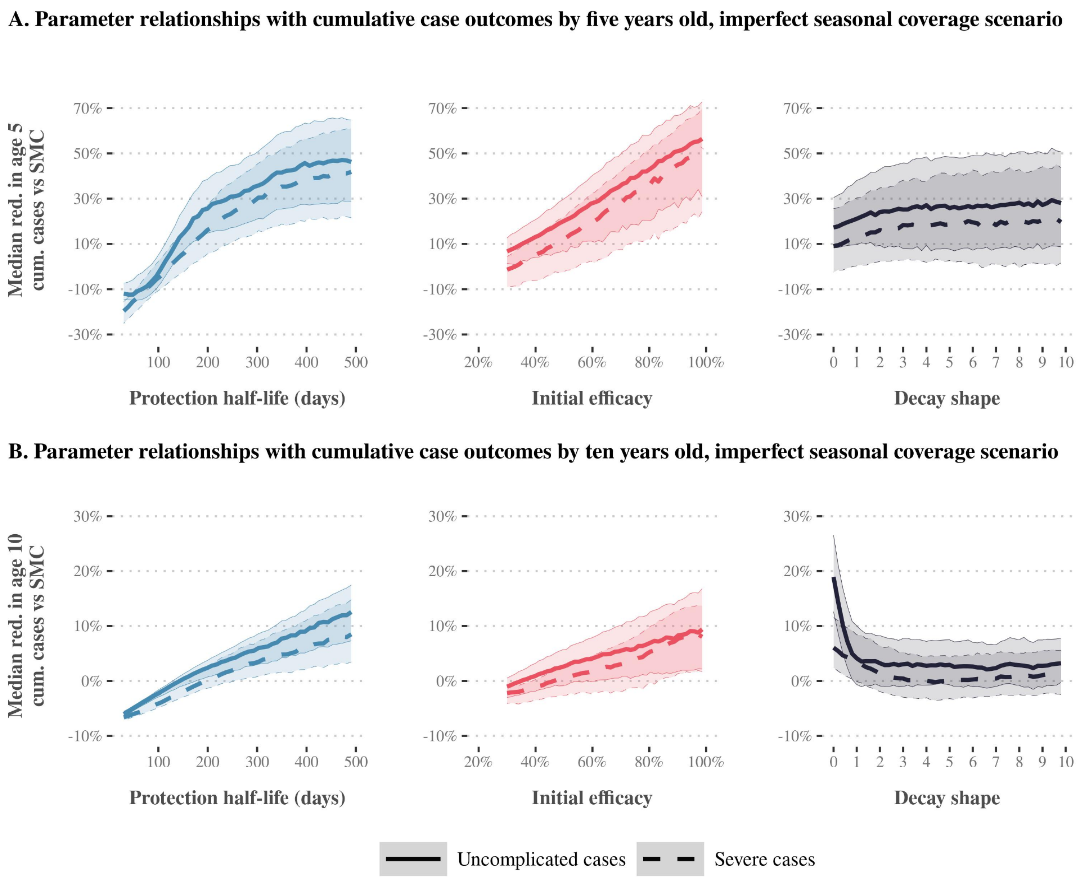

Fig G: Gaussian Process regression emulator predictions for the relationship between pre-liver stage product properties and expected reductions in cumulative uncomplicated and severe cases relative to SMC alone, evaluated with imperfect seasonal coverage of SMC

Results show the imperfect seasonal coverage scenario, where children aged three to 59 months received four SMC cycles. Transmission is high (32% *Pf*PR$_{2-10}$), 75% of malaria cases occur within six months of the year, and the probability of seeking first-line treatment for clinical malaria over 14 days is low (10%). Each panel shows the median reduction in cumulative uncomplicated (solid lines) or severe cases (dashed lines) achieved by combining seasonal deployment of a pre-liver stage product with SMC, relative to cumulative cases when SMC is deployed alone. Median reductions are calculated by dividing the parameter range for the product property shown on the x-axis (protection half-life, initial efficacy, decay shape) into 51 segments, and calculating the median outcome for each given segment across all other parameter values. Shaded regions represent the 25% and 75% quantiles of the corresponding reductions. Emulator predictions are shown separately for cumulative case outcomes at five (panels A) and ten years old (panels B).

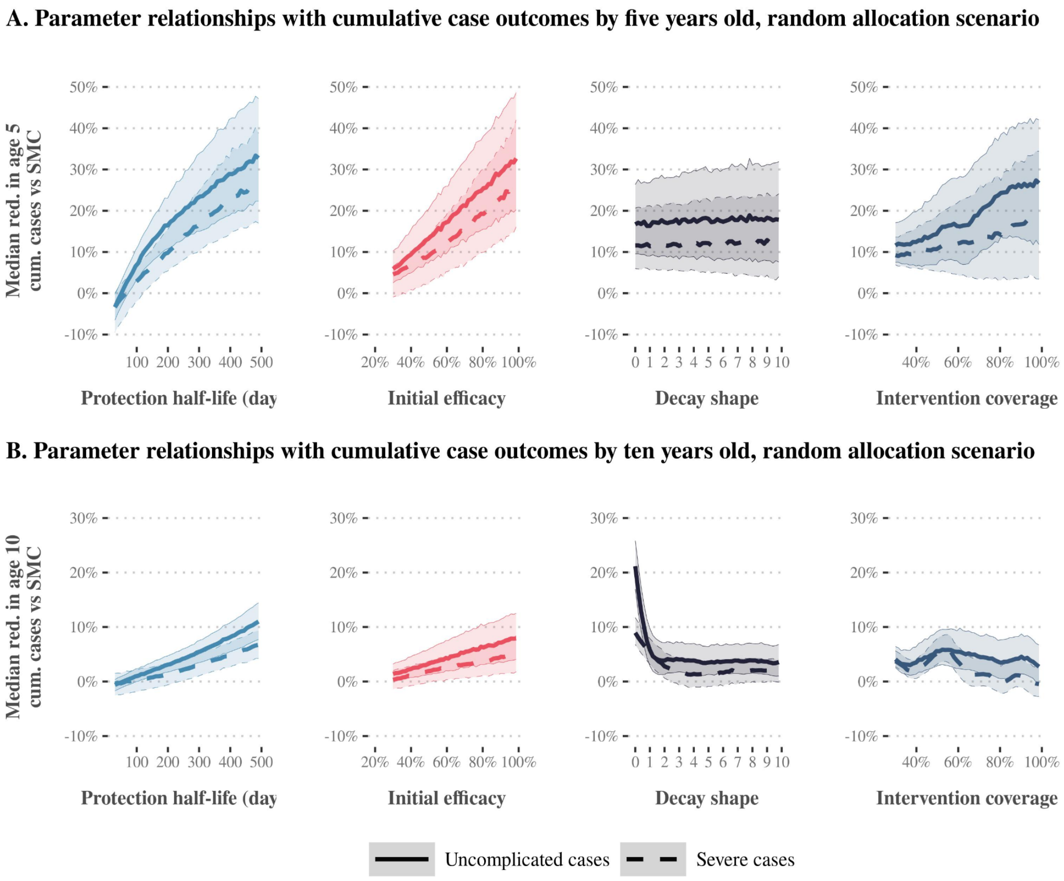

Fig H: Gaussian Process regression emulator predictions for the relationship between pre-liver stage product properties and expected reductions in cumulative uncomplicated and severe cases relative to SMC alone, evaluated with random allocation of SMC cycles

Results show the random allocation scenario, where between 30% and 100% of children aged three to 59 months are randomly allocated to receive five SMC cycles. Transmission is high (32% *Pf*PR$_{2\text{-}10}$), 75% of malaria cases occur within six months of the year, and the probability of seeking first-line treatment for clinical malaria over 14 days is low (10%). Each panel shows the median reduction in cumulative uncomplicated (solid lines) or severe cases (dashed lines) achieved by combining seasonal deployment of a pre-liver stage product with SMC, relative to cumulative cases when SMC is deployed alone. Median reductions are calculated by dividing the parameter range for the product property shown on the x-axis (protection half-life, initial efficacy, decay shape) into 51 segments, and calculating the median outcome for each given segment across all other parameter values. Shaded regions represent the 25% and 75% quantiles of the corresponding reductions. Emulator predictions are shown separately for cumulative case outcomes at five (panels A) and ten years old (panels B).

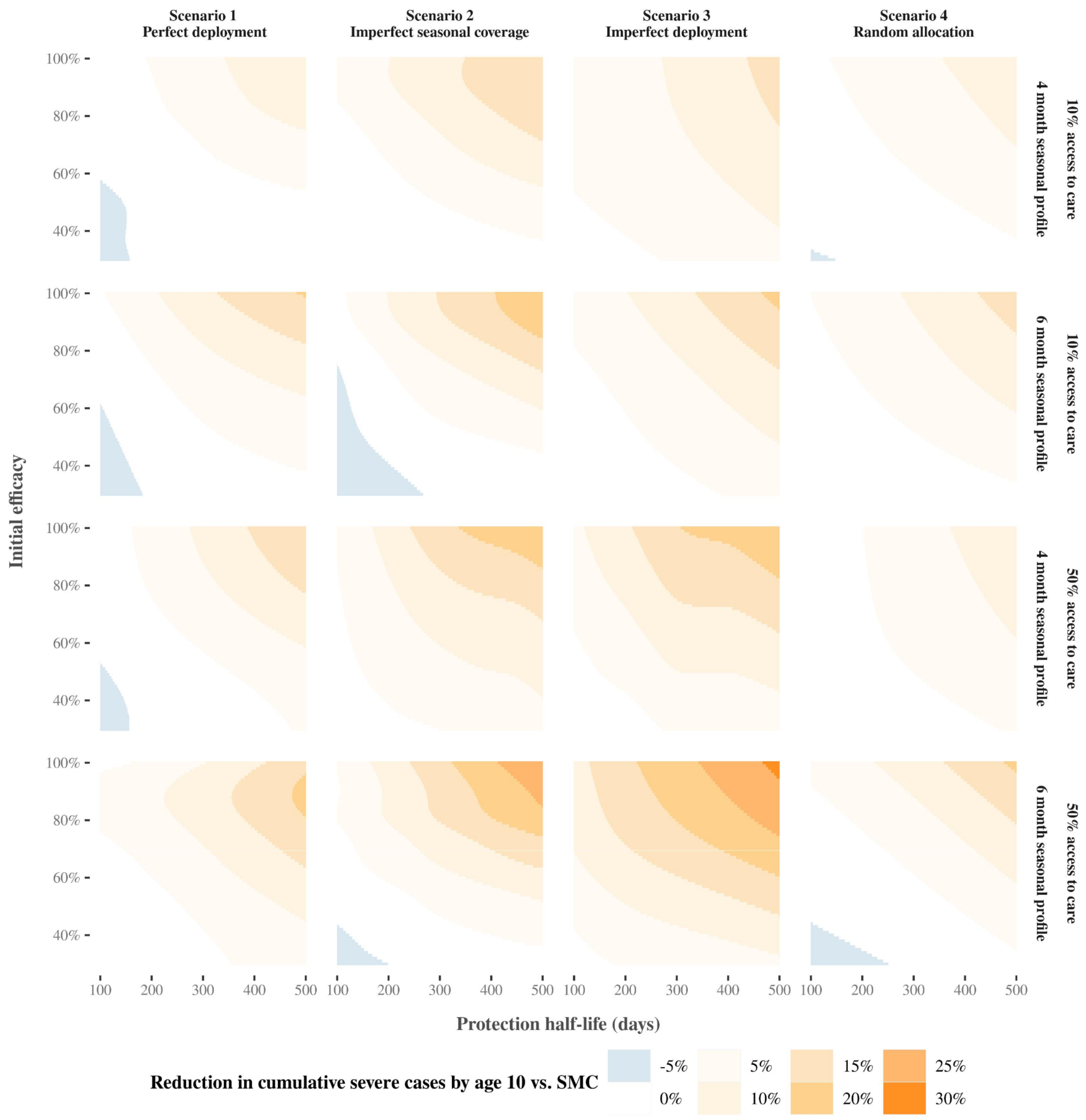

Fig I: Predicted relationships between pre-liver stage protection half-life, initial efficacy and reductions in cumulative severe cases by ten years old, compared across settings and deployment scenarios for a product with a long tail of protection

Each square in each grid indicates the predicted reduction if a pre-liver stage product with the given protection half-life (x-axis) and initial efficacy (y-axis) were deployed in addition to SMC, assuming a long tail of protection (decay

shape parameter 0.7). Reductions are rounded to the nearest 5%. Results show a scenario where transmission is high (ranging between 19% and 33% *Pf*PR$_{2-10}$ depending on the seasonality profile and level of access to care). Panels depict predicted relationships for four different SMC deployment scenarios, two different seasonality profiles, and two different levels of access to first-line treatment for clinical malaria over 14 days (access to care).

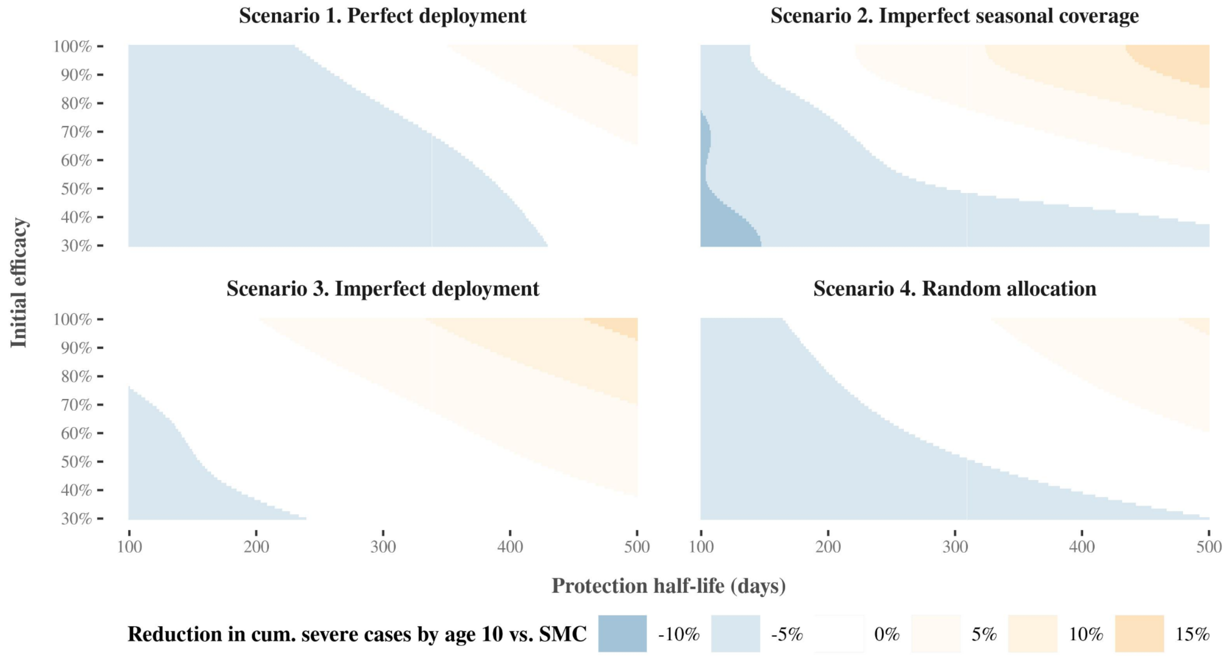

Fig J: Predicted relationships between pre-liver stage protection half-life, initial efficacy and reductions in cumulative severe cases by ten years old, compared across SMC deployment scenarios for a product with a rapid decay in protection

Each square in each grid indicates the predicted reduction if a pre-liver stage product with the given protection half-life (x-axis) and initial efficacy (y-axis) were deployed in addition to SMC, assuming a rapid decay in protection (decay shape parameter 4). Reductions are rounded to the nearest 5%. Results show a scenario where transmission is high (32% *Pf*$PR_{2\text{-}10}$), 75% of malaria cases occur within six months of the year, and the probability of seeking first-line treatment for clinical malaria over 14 days is low (10%). Panels depict predicted relationships for four different SMC deployment scenarios.

3. Additional results: Combining SMC with seasonal deployment of a blood stage product

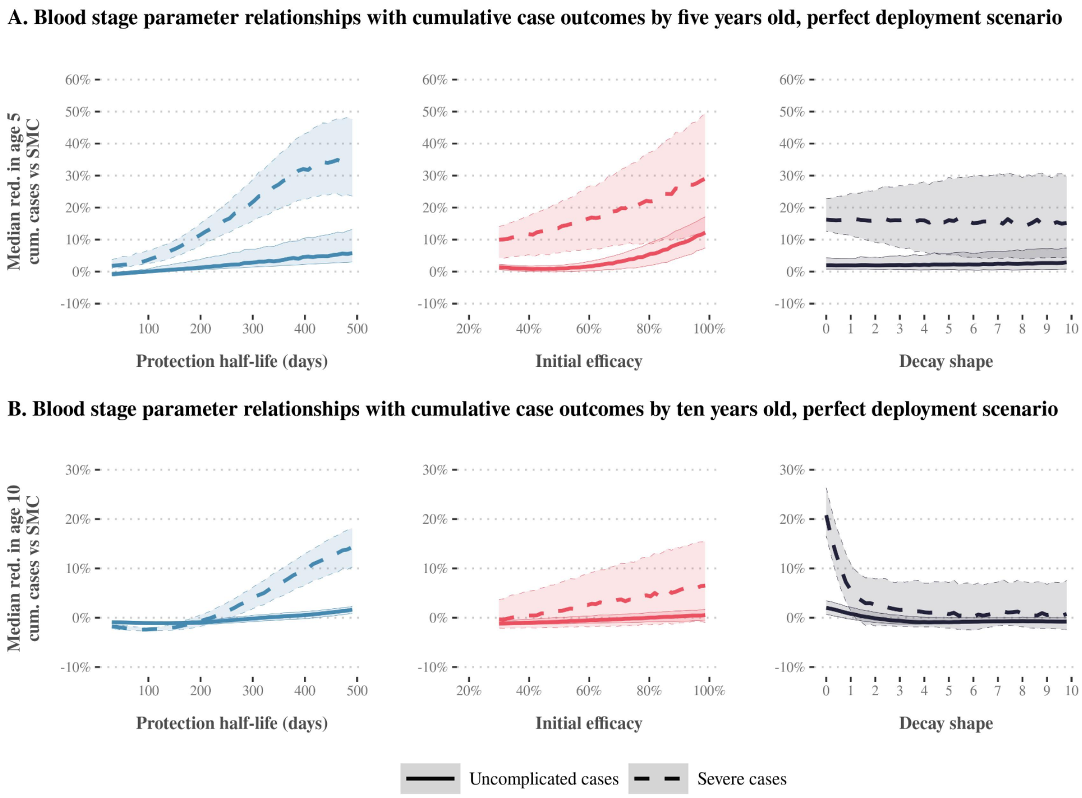

Fig K: Gaussian Process regression emulator predictions for the relationship between blood stage product properties and expected reductions in cumulative uncomplicated and severe cases relative to SMC alone, evaluated with perfect coverage of SMC

Results show the perfect deployment scenario, where children aged three to 59 months received five SMC cycles. Transmission is high (32% *Pf*PR$_{2-10}$), 75% of malaria cases occur within six months of the year, and the probability of seeking first-line treatment for clinical malaria over 14 days is low (10%). Each panel shows the median reduction in cumulative uncomplicated (solid lines) or severe cases (dashed lines) achieved by combining seasonal deployment of a blood stage product with SMC, relative to cumulative cases when SMC is deployed alone. Median reductions are calculated by dividing the parameter range for the product property shown on the x-axis (protection half-life, initial efficacy, decay shape) into 51 segments, and calculating the median outcome for each given segment across all other parameter values. Shaded regions represent the 25% and 75% quantiles of the corresponding reductions. Emulator predictions are shown separately for cumulative case outcomes at five (panels A) and ten years old (panels B).

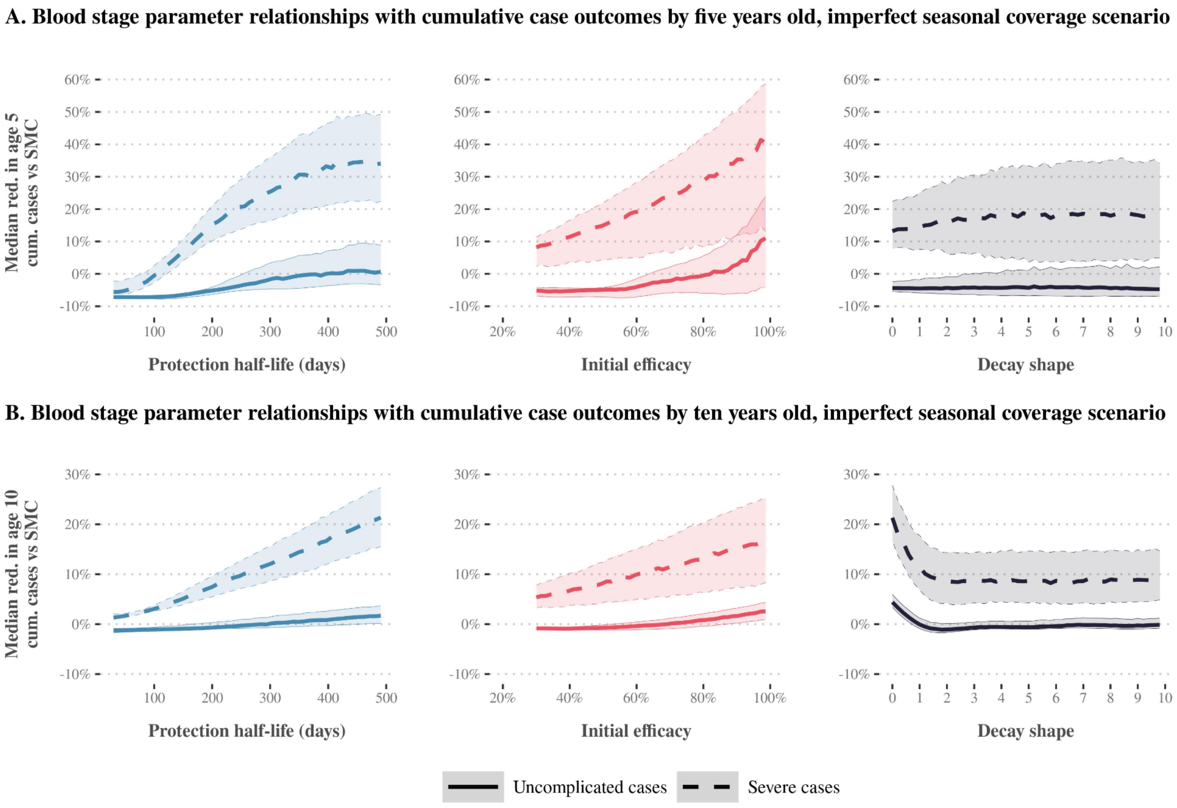

Fig L: Gaussian Process regression emulator predictions for the relationship between blood stage product properties and expected reductions in cumulative uncomplicated and severe cases relative to SMC alone, evaluated with imperfect seasonal coverage of SMC

Results show the imperfect seasonal coverage scenario, where children aged three to 59 months received four SMC cycles. Transmission is high (32% *Pf*$PR_{2\text{-}10}$), 75% of malaria cases occur within six months of the year, and the probability of seeking first-line treatment for clinical malaria over 14 days is low (10%). Each panel shows the median reduction in cumulative uncomplicated (solid lines) or severe cases (dashed lines) achieved by combining seasonal deployment of a blood stage product with SMC, relative to cumulative cases when SMC is deployed alone. Median reductions are calculated by dividing the parameter range for the product property shown on the x-axis (protection half-life, initial efficacy, decay shape) into 51 segments, and calculating the median outcome for each given segment across all other parameter values. Shaded regions represent the 25% and 75% quantiles of the corresponding reductions. Emulator predictions are shown separately for cumulative case outcomes at five (panels A) and ten years old (panels B).

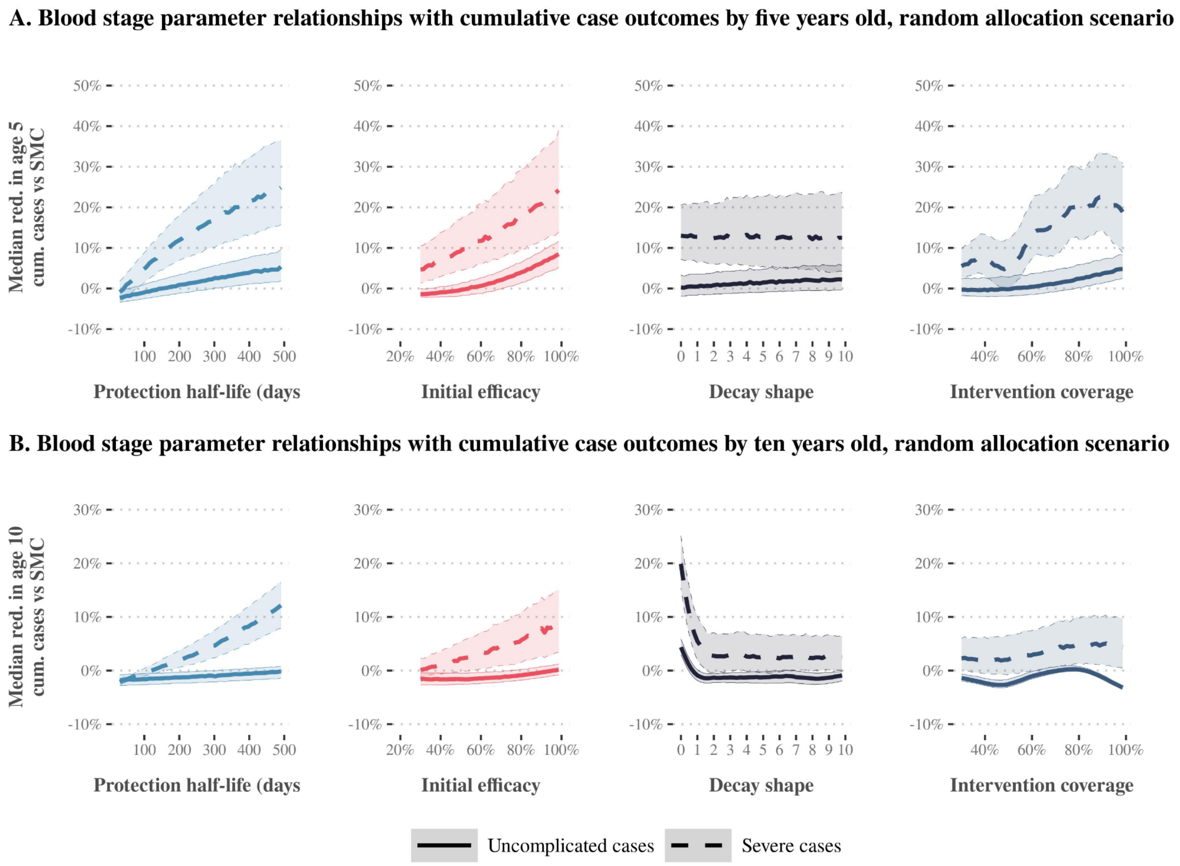

Fig M: Gaussian Process regression emulator predictions for the relationship between blood stage product properties and expected reductions in cumulative uncomplicated and severe cases relative to SMC alone, evaluated with random allocation of SMC cycles

Results show the random allocation scenario, where between 30% and 100% of children aged three to 59 months are randomly allocated to receive five SMC cycles. Transmission is high (32% *Pf*PR_{2-10}), 75% of malaria cases occur within six months of the year, and the probability of seeking first-line treatment for clinical malaria over 14 days is low (10%). Each panel shows the median reduction in cumulative uncomplicated (solid lines) or severe cases (dashed lines) achieved by combining seasonal deployment of a blood stage product with SMC, relative to cumulative cases when SMC is deployed alone. Median reductions are calculated by dividing the parameter range for the product property shown on the x-axis (protection half-life, initial efficacy, decay shape) into 51 segments, and calculating the median outcome for each given segment across all other parameter values. Shaded regions represent the 25% and 75% quantiles of the corresponding reductions. Emulator predictions are shown separately for cumulative case outcomes at five (panels A) and ten years old (panels B).

4. Additional results: Combining SMC with seasonal deployment of a multi-stage product

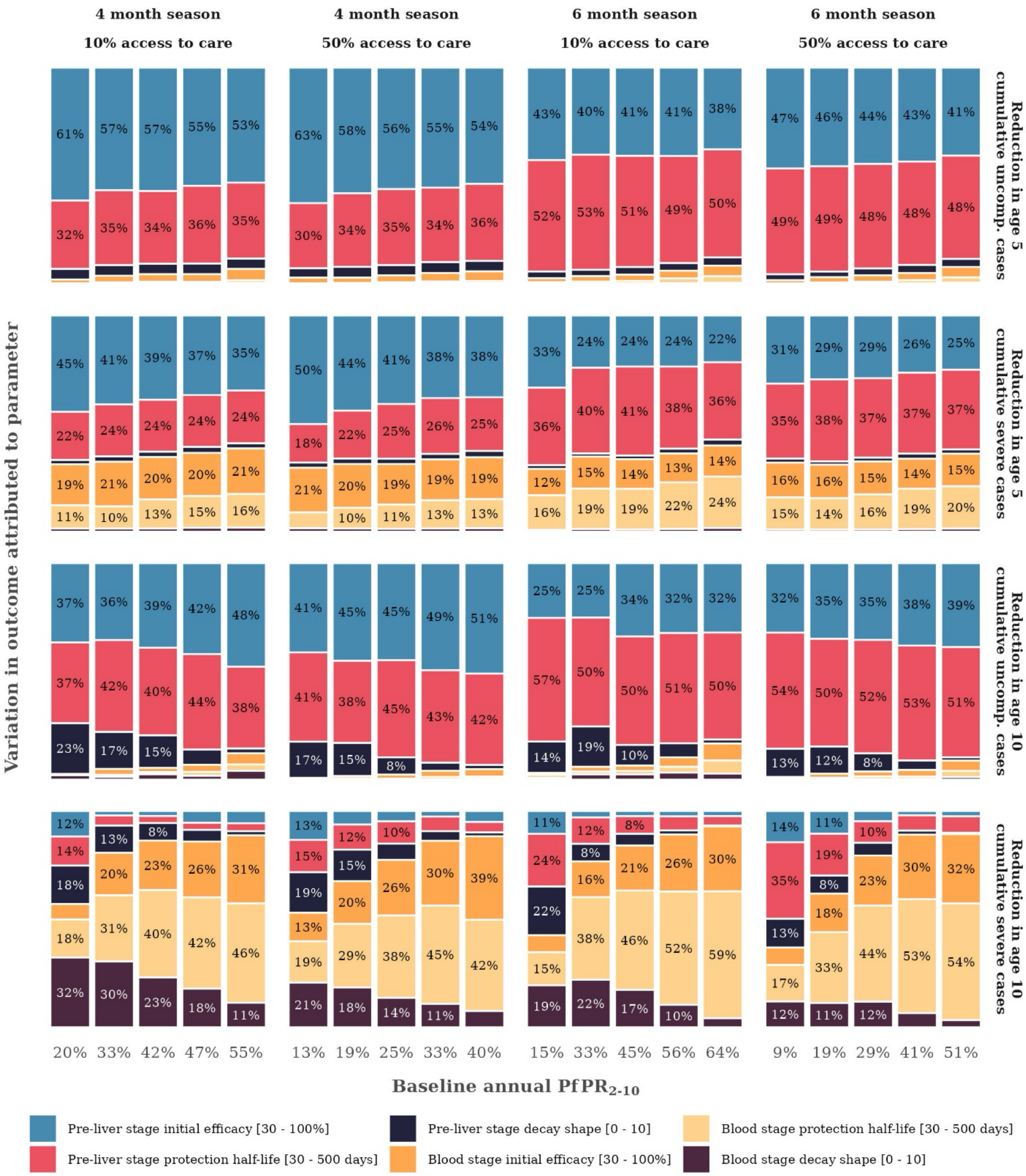

Fig N: Drivers of impact on all cumulative case outcomes for a product with both pre-liver stage and blood stage activity deployed together with three yearly cycles of SMC, compared with SMC deployed alone

Bars show the total Sobol effect indices for intervention model parameters. Total Sobol effect indices can be interpreted as the proportion of variation in the cumulative case outcome that can be attributed to a small change in each model parameter and its interactions with other parameters. Indices are shown across levels of transmission intensity (x-axis) for a scenario where three yearly cycles of SMC are deployed (imperfect coverage scenario).

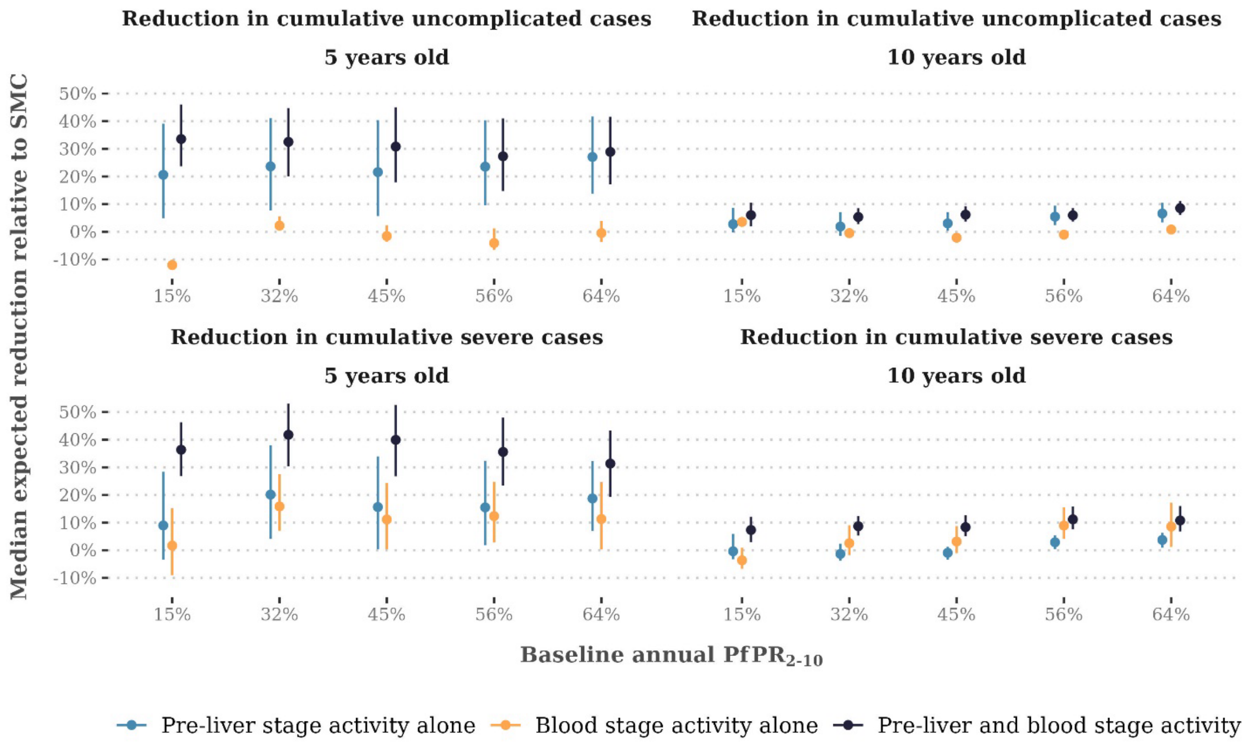

Fig O: Differences in median impact on cumulative case outcomes for products with pre-liver stage activity alone, blood stage activity alone, and multi-stage pre-liver stage and blood stage activity, evaluated relative to perfect deployment of SMC

Results show the perfect deployment scenario, where children aged three to 59 months received five SMC cycles. 75% of malaria cases occur within six months of the year and the probability of seeking first-line treatment for clinical malaria over 14 days is low (10%). Points in each panel show the median expected reduction in cumulative cases achieved by combining seasonal deployment of a pre-liver stage, blood stage, or multi-stage product with SMC, relative to cumulative cases when SMC is deployed alone. Median reductions are evaluated across all possible combinations of product initial efficacy, protection half-life, and decay shape. Bars indicate the 25% and 75% quantiles of the corresponding reductions.

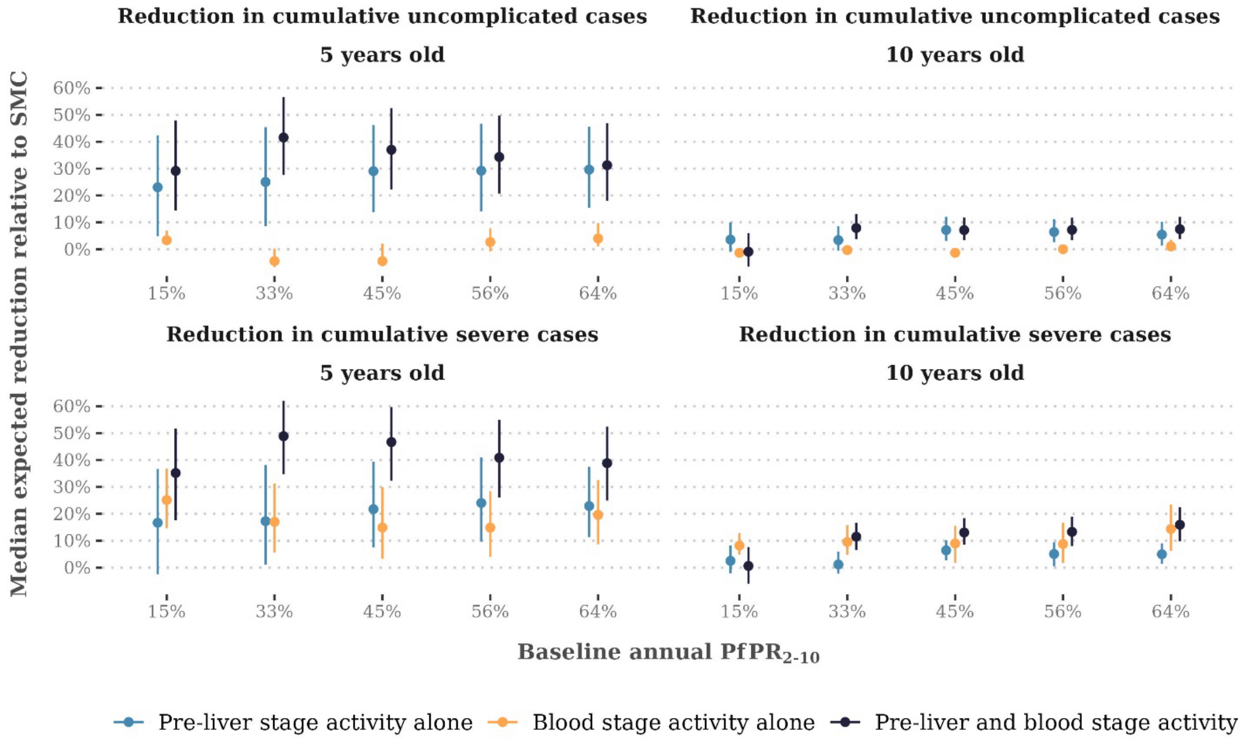

Fig P: Differences in median impact on cumulative case outcomes for products with pre-liver stage activity alone, blood stage activity alone, and multi-stage pre-liver stage and blood stage activity, evaluated relative to imperfect seasonal coverage of SMC

Results show the imperfect seasonal coverage scenario, where children aged three to 59 months received four SMC cycles. 75% of malaria cases occur within six months of the year and the probability of seeking first-line treatment for clinical malaria over 14 days is low (10%). Points in each panel show the median expected reduction in cumulative cases achieved by combining seasonal deployment of a pre-liver stage, blood stage, or multi-stage product with SMC, relative to cumulative cases when SMC is deployed alone. Median reductions are evaluated across all possible combinations of product initial efficacy, protection half-life, and decay shape. Bars indicate the 25% and 75% quantiles of the corresponding reductions.

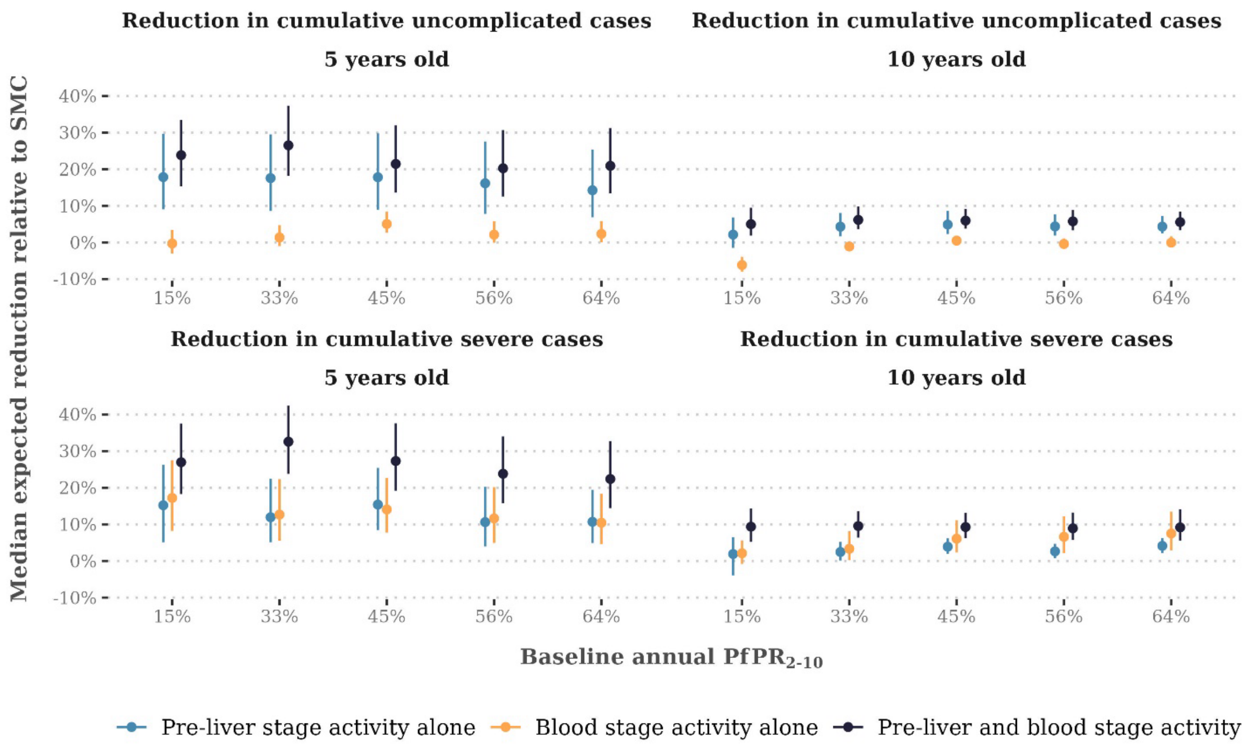

Fig Q: Differences in median impact on cumulative case outcomes for products with pre-liver stage activity alone, blood stage activity alone, and multi-stage pre-liver stage and blood stage activity, evaluated relative to random coverage of SMC cycles

Results show the random allocation scenario, where between 30% and 100% of children aged three to 59 months were randomly allocated to receive each of five SMC cycles. 75% of malaria cases occur within six months of the year and the probability of seeking first-line treatment for clinical malaria over 14 days is low (10%). Points in each panel show the median expected reduction in cumulative cases achieved by combining seasonal deployment of a pre-liver stage, blood stage, or multi-stage product with SMC, relative to cumulative cases when SMC is deployed alone. Median reductions are evaluated across all possible combinations of product initial efficacy, protection half-life, decay shape, and SMC cycle coverage. Bars indicate the 25% and 75% quantiles of the corresponding reductions.

5. References

1. Smith TA, Killeen GF, Maire N, Ross A, Molineaux L, Tediosi F, et al. Mathematical modeling of the impact of malaria vaccines on the clinical epidemiology and natural history of Plasmodium falciparum malaria: Overview. Am J Trop Med Hyg. 2006 Aug;75(2 Suppl):1–10.

2. Reiker T, Golumbeanu M, Shattock A, Burgert L, Smith TA, Filippi S, et al. Emulator-based Bayesian optimization for efficient multi-objective calibration of an individual-based model of malaria. Nat Commun. 2021 Dec 10;12(1):7212.

3. Smith TA, Maire N, Dietz K, Killeen GF, Vounatsou P, Molineaux L, et al. Relationship between the entomologic inoculation rate and the force of infection for Plasmodium falciparum malaria. Am J Trop Med Hyg. 2006 Aug;75(2 Suppl):11–8.

4. Collins WE, Jeffery GM. A retrospective examination of the patterns of recrudescence in patients infected with Plasmodium falciparum. Am J Trop Med Hyg. 1999 Jul;61(1 Suppl):44–8.

5. Ross A, Killeen GF, Smith TA. Relationships between host infectivity to mosquitoes and asexual parasite density in Plasmodium falciparum. Am J Trop Med Hyg. 2006 Aug;75(2):32–7.

6. Killeen GF, Ross A, Smith TA. Infectiousness of malaria-endemic human populations to vectors. Am J Trop Med Hyg. 2006 Aug;75(2 Suppl):38–45.

7. Ross A, Maire N, Molineaux L, Smith TA. An epidemiologic model of severe morbidity and mortality caused by Plasmodium falciparum. Am J Trop Med Hyg. 2006 Aug;75(2 Suppl):63–73.

8. Ross A, Smith TA. The effect of malaria transmission intensity on neonatal mortality in endemic areas. Am J Trop Med Hyg. 2006 Aug;75(2 Suppl):74–81.

9. Smith TA, Ross A, Maire N, Rogier C, Trape JF, Molineaux L. An epidemiologic model of the incidence of acute illness in Plasmodium falciparum malaria. Am J Trop Med Hyg. 2006 Aug;75(2 Suppl):56–62.

10. Ekstrom AM, Clark J, Byass P, Lopez A, De Savigny D, Moyer CA, et al. INDEPTH Network: contributing to the data revolution. Lancet Diabetes Endocrinol. 2016 Feb;4(2):97.

11. Stuckey EM, Smith TA, Chitnis N. Seasonally dependent relationships between indicators of malaria transmission and disease provided by mathematical model simulations. PLOS Comput Biol. 2014 Sep;10(9):e1003812.

12. Tediosi F, Maire N, Smith TA, Hutton G, Utzinger J, Ross A, et al. An approach to model the costs and effects of case management of Plasmodium falciparum malaria in sub-saharan Africa. Am J Trop Med Hyg. 2006 Aug;75(2 Suppl):90–103.

13. Chitnis N, Hardy D, Smith TA. A periodically-forced mathematical model for the seasonal dynamics of malaria in mosquitoes. Bull Math Biol. 2012 May;74(5):1098–124.

14. Penny MA, Maire N, Studer A, Schapira A, Smith TA. What should vaccine developers ask? Simulation of the effectiveness of malaria vaccines. PLOS ONE. 2008 Sep 11;3(9):e3193.

15. Smith TA, Ross A, Maire N, Chitnis N, Studer A, Hardy D, et al. Ensemble modeling of the likely public health impact of a pre-erythrocytic malaria vaccine. PLOS Med. 2012 Jan;9(1):e1001157.

16. Golumbeanu M, Yang GJ, Camponovo F, Stuckey EM, Hamon N, Mondy M, et al. Leveraging mathematical models of disease dynamics and machine learning to improve development of novel malaria interventions. Infect Dis Poverty. 2022;11(61).

17. Braunack-Mayer L, Malinga J, Masserey T, Nekkab N, Sen S, Schellenberg D, et al. Design and selection of drug properties to increase the public health impact of next-generation seasonal malaria chemoprevention: a modelling study. Lancet Glob Health. 2024 Mar 1;12(3):e478–90.

18. Datoo MS, Dicko A, Tinto H, Ouédraogo JB, Hamaluba M, Olotu A, et al. Safety and efficacy of malaria vaccine candidate R21/Matrix-M in African children: a multicentre, double-blind, randomised, phase 3 trial. The Lancet. 2024 Feb 10;403(10426):533–44.

19. Dicko A, Ouedraogo JB, Zongo I, Sagara I, Cairns M, Yerbanga RS, et al. Seasonal vaccination with RTS,S/AS01(E) vaccine with or without seasonal malaria chemoprevention in children up to the age of 5 years in Burkina Faso and Mali: a double-blind, randomised, controlled, phase 3 trial. Lancet Infect Dis. 2024 Jan;24(1):75–86.

20. Binois M, Gramacy RB. hetGP: Heteroskedastic Gaussian Process Modeling and Sequential Design in R. J Stat Softw. 2021 Jul;98(13):1–44.

21. Burgert L, Reiker T, Golumbeanu M, Moehrle JJ, Penny MA. Model-informed target product profiles of long-acting-injectables for use as seasonal malaria prevention. PLOS Glob Public Health. 2022;2(3):e0000211.

22. Iooss B, Da Veiga S, Janon A, Pujol G. Global sensitivity analysis of model outputs. R; 2021.

23. Sobol IM. Global sensitivity indices for nonlinear mathematical models and their Monte Carlo estimates. Math Comput Sim. 2001;55(1–3):271–80.

24. R Core Team. R: A language and environment for statistical computing [Internet]. Vienna, Austria: R Foundation for Statistical Computing; 2024. Available from: https://www.R-project.org/
