# Supplementary material for: Combining seasonal malaria chemoprevention with novel therapeutics for malaria prevention: a mathematical modelling study
Source: PLoS Comput Biol. 2026 Feb 26;22(2):e1014021. doi: 10.1371/journal.pcbi.1014021 (PMC12962535; doi:10.1371/journal.pcbi.1014021)
Supplement: Attachment — Submitted filename: PCOMPBIOL-D-25-01802_report.pdf [file pcbi.1014021.s002.pdf]

Title:

Combining seasonal malaria chemoprevention with novel therapeutics for malaria prevention: a mathematical modelling study

Authors:

Lydia Braunack-Mayer[1,2], Josephine Malinga[1,3], Narimane Nekkab[4,5], Sherrie L Kelly[4], Jörg J Möhrle[4,5], Melissa A Penny[1,3,*]

Affiliations:

1 The Kids Research Institute Australia, Nedlands, WA, Australia

2 Institute of Social and Preventive Medicine, University of Bern, Bern, Switzerland

3 Centre for Child Health Research, The University of Western Australia, Crawley, WA, Australia

4 Swiss Tropical and Public Health Institute, Allschwil, Switzerland

5 University of Basel, Basel, Switzerland

Reviewer Summary:

This study sought to provide evidence on the potential benefit of combining novel malaria therapeutics with SMC activities. An individual based transmission model was used to estimate the public health impact of combining SMC with new therapeutics to reduce uncomplicated and severe cases during and five years after deployment. Therapeutics targeting pre-liver and/or blood stages were characterised based on initial efficacy, duration of efficacy, and protection half-life to identify suitable candidates required to achieve target reductions in infections among children under 10. The model simulates a cohort of children born at the beginning of intervention to measure reductions in uncomplicated and severe cases under four deployment scenarios. Three key observations were made in the study, firstly, a therapeutic with >50% initial efficacy and >230 days protection half-life led to >23% reduction in severe cases during, and >5% five years after intervention stopped in a high transmission setting with three cycles of SMC. Secondly, therapeutics with longer protection provide additional benefit in regions with four or five SMC cycles. And thirdly, multi-stage therapeutics showed substantial public health impact, particularly in regions where SMC efficacy is compromised. As a result, it is concluded that therapeutics targeting both pre-liver and blood stages need to be prioritised for development and selected for combination with SMC.

Recommendation

The study uses a mathematical model to address the challenge of adherence to SMC based on data from an RCT for combining SMC with a vaccine, phase II trial on monoclonal antibodies, and the relative effectiveness of long-acting injectables.

Utilising an established model such as OpenMalaria, which has been validated on historical data added credibility to model outcomes. Furthermore, calibrating vaccine booster efficacy and properties to clinical trial data provided a reasonable baseline for impact.

Simulation results explored the impact of pre-liver stage, blood stage, and multistage therapeutics, which enabled a comprehensive analysis into the potential benefits of combining SMC with novel therapeutics.

Therefore, I strongly recommend that this manuscript be **accepted** for publication.

Minor Issues

1. Lines 28 – 29 should rather be rewritten as "... to reduce cumulative severe cases by >23% during interventions, and >5% five years after deployment stopped.", for clarity.
2. Lines 304 – 311 do not use the terms used in Figure 4. This makes it difficult for the reader to follow without referring to the caption of Figure 1 (lines 201 – 205), where the scenarios are clearly defined in terms of the SMC cycles, coverage, etc. How the results are currently presented does not seem to link with Figure 4, so the authors should rephrasing these lines for clarity and provide a direct link with the results presented in the image.
3. The attributed percentage is 95% for uncomplicated cases, and the 70% mentioned in lines 345 – 347 is for severe cases. Also, this is particularly measured at five years old, so the text should reflect that.
4. The authors should consider mentioning the baseline annual prevalence as the variations at ten years old differ distinctly at 15% and 64% annual prevalence for severe cases.

Comments

Introduction:

The lack of adherence to SMC and the threat of parasite resistance to SP-AQ have prompted a need for novel therapeutics to be used in combination with SMC.

A Randomised Control Trial (RCT) was conducted to evaluate the benefits of combining SMC with a vaccine. SP-AQ clears blood stage and prevents pre-liver emergence, while the RTS,S vaccine prevents liver stage infection. This combination was found to be more effective than either intervention alone. However, it is not yet known whether similar benefits can be observed for other therapeutics.

The first phase II results from pre-liver stage monoclonal antibodies are available. These show seasonal suitability with high levels of protection following single dose, but cost-effectiveness concerns remain.

Atovaquone-proguanil injectables offer three to four months of protection at lower cost than monoclonal antibodies and target multiple stages.

These three therapeutics act differently at pre-liver and blood stages, and how they can improve the benefits of SMC is important to know, so an individual based transmission model is used to address this.

Materials & Methods:

Malaria transmission model

OpenMalaria is an established model with a detailed framework that has been validated on historical data. It has multiple components to cater for different stages of the parasite lifecycle. Hence, it was appropriate for this research study.

Although immunity is part of the OpenMalaria framework, it is not modelled in the study. The authors have acknowledged this as a limitation of their research, and future studies are urged to validate changes in model outcomes when immunity is accounted for.

Intervention dynamics and deployment

The activity of each therapeutic at various stages is modelled independently, so any synergistic effects emerge as a result of the OpenMalaria model structure.

LHS is a statistical sampling method used to estimate parameter values and a table of sampled parameter ranges is provided for reproducibility.

Intervention model validation

Bayesian optimisation was used to calibrate vaccine booster efficacy and properties to data from a clinical trial. The authors claim that this process was able to reproduce the observed monthly incidence despite not providing evidence of this.

Results:

Figure 2 shows the outcomes of combining SMC with a pre-liver stage therapeutic

Two therapeutics were considered, and reductions were observed by age five when the intervention stops. Long duration, long tails, and high efficacy were crucial to maintaining reductions in infections. Also, higher efficacy could be preferred for shorter durations to achieve the same long-term benefits 5 years after intervention has stopped. However, longer duration is required to benefit in settings with high SMC coverage.

Figure 5 shows the outcomes of combining SMC with a seasonal blood stage therapeutic

Blood stage therapeutics primarily serve to supress disease severity rather than prevent infections. High initial efficacy is required to impact infections in the long-term but reductions in severe cases could be achieved with lower efficacy.

Figure 6 shows the outcomes of combining SMC with a multistage therapeutic

Pre-liver activity of multistage therapeutics is attributed to substantial reductions in infections during intervention. While long term reductions in severe cases are attributed to blood stage activity after intervention has stopped. It is concluded that multistage therapeutics provide greater impact than single stage ones, particularly pre-liver stages the benefits of which are greatly improved.

Discussion:

Pre-liver stage vaccines are proposed as candidates to partner with SMC to reduce uncomplicated cases.

Extended pre-liver or multistage activity is required to sustain benefits throughout childhood.

Multistage therapeutics are further proposed for selection over single stage therapeutics.

Integrating immunity into the model would have implications for outcomes related to multistage therapeutics because blood stage activity provides protections against parasites that escape pre-liver stage protection.

Due to the paucity of data on clinical trials as mentioned in the discussion, the authors suggest that the development of multistage therapeutics, and more specifically, blood stage active long-acting injectables could fill this gap.

Layering SMC with a vaccine or monoclonal antibodies would benefit the greatest in regions where SMC coverage is compromised, either due to lack of adherence or parasite resistance. In regions where coverage is high, then long-tailed therapeutics are needed to benefit from combinations with SMC. Given the cost implications of four or five SMC cycles, these trade-offs beg the question of the cost-effectiveness of combining therapeutics with SMC at different coverage levels. However, this was not modelled in the study due to uncertainties related to the costs of developing new therapeutics.

Since this study focused on a high transmission areas and children under 10 years old, the authors suggest that future modelling studies should explore these combination strategies in perennial settings and target specific age groups.

Multistage therapeutics are recommended for combination with SMC to maintain benefits in children, 5 years after intervention has stopped.
