# Supplementary material for: Combining seasonal malaria chemoprevention with novel therapeutics for malaria prevention: a mathematical modelling study
Source: PLoS Comput Biol. 2026 Feb 26;22(2):e1014021. doi: 10.1371/journal.pcbi.1014021 (PMC12962535; doi:10.1371/journal.pcbi.1014021)
Supplement: Attachment — Submitted filename: Response to Reviewers.pdf [file pcbi.1014021.s004.pdf]

THE UNIVERSITY OF WESTERN AUSTRALIA

Prof Melissa Penny
Fiona Stanley Chair in Child Health Research
UWA Centre for Child Health Research
melissa.penny@uwa.edu.au

22 January 2026

Response to editorial staff and reviewers for:

Combining seasonal malaria chemoprevention with novel therapeutics for malaria prevention: a mathematical modelling study

Lydia Braunack-Mayer[1, 2], *Josephine Malinga*[1,3], *Narimane Nekkab*[4, 5], *Sherrie L Kelly*[4], *Jörg J Möhrle*[4, 5], *Melissa A Penny*[1, 3]

1 The Kids Research Institute Australia, Nedlands, WA, Australia
2 Institute of Social and Preventive Medicine, University of Bern, Bern, Switzerland
3 Centre for Child Health Research, The University of Western Australia, Crawley, WA, Australia
4 Swiss Tropical and Public Health Institute, Allschwil, Switzerland
5 University of Basel, Basel, Switzerland

Correspondence to Prof Melissa A Penny, University of Western Australia, Crawley, Australia, melissa.penny@uwa.edu.au

Submission ID PCOMPBIOL-D-25-01802

Dear Dr Flegg,

On behalf of our co-authors, we are pleased to submit the revised version of our manuscript titled *"Combining seasonal malaria chemoprevention with novel therapeutics for malaria prevention: a mathematical modelling study"* together with our response to our reviewers.

Our sincere thanks for your comments and for giving us an opportunity to revise our manuscript. We are particularly grateful to our reviewers for their valuable and constructive feedback; their comments have helped to strengthen our perspective and improve the clarity of our manuscript.

We have provided a point-by-point response to each comment below. Reviewer comments are in black text, and our responses are in blue. For each comment, we have summarised changes to the main manuscript and point to the revised text in the manuscript. We have also provided additional information for several responses below, to provide further information to our editor and reviewers.

Please do not hesitate to ask if you have any questions about our revised manuscript. We are looking forward to hearing from you soon.

Yours sincerely,

Prof Melissa Penny and Dr Lydia Braunack-Mayer, on behalf of the authors

Prof Melissa Penny
Fiona Stanley Chair in Child Health Research
UWA Centre for Child Health Research
melissa.penny@uwa.edu.au

Reviewer #1

The authors should calculate the R0 for the different intervention scenarios as well.

Thank you very much for your review and for this suggestion. While R_0 is useful for describing the transmission intensity of an infectious disease in compartmental models, its standard definition relies on a set of simplifying assumptions: populations are fully susceptible, mix evenly and have unlimited opportunities to infect others. The model we have used in this study is necessarily more complex than this and, as a result, R_0 cannot be calculated using standard approaches.

OpenMalaria is a stochastic, individual-based model in which individuals can experience different mosquito biting rates, develop different levels of immunity over time, and respond differently to interventions. Transmission is therefore age-dependent, seasonal, and driven by stochastic processes rather than a single average mechanism. As explained in detail in Griffin's 2016 paper, these model characteristics mean that defining R_0 within OpenMalaria is conceptually and mathematically difficult even in the absence of immunity and interventions. Once immunity acquisition, seasonality, and intervention effects are included, as would be required for our study, deriving an expression for R_0 would require substantial additional methodological development and would constitute multiple separate studies.

For malaria modelling studies, transmission intensity is therefore more commonly characterised using measures such as the entomological inoculation rate (EIR), simulated incidence, or parasite prevalence, which are directly observed in epidemiological studies and clinical trials. Importantly, unlike R_0, these measures can be both represented in and extracted from individual-based models and carry clear biological meaning. For these reasons, and consistent with standard practice in malaria modelling, we use *Plasmodium falciparum* prevalence in children aged 2-10 years as our measure of transmission intensity and calculate intervention impact on the incidence of uncomplicated and severe malaria.

Reference: Griffin J. Malaria Journal. 2016. https://doi.org/10.1186/s12936-016-1437-9

Prof Melissa Penny
Fiona Stanley Chair in Child Health Research
UWA Centre for Child Health Research
melissa.penny@uwa.edu.au

Reviewer 2

The reviewer thanks the authors for an excellent manuscript, presenting relevant findings on profiles for new therapeutics when combined with SMC based on a mathematical modeling study. There are only a few questions and minor comments for the authors to address, listed below.

The manuscript describes results from a mathematical modeling study that evaluated the health benefits of SMC in combination with new therapeutics (vaccines, monoclonal antibodies, and long-acting injectables), targeting multiple stages of the parasite life cycle, across hypothetical transmission settings during and 5 years after interventions, targeted to children under the age of five years, stopped. The author's results highlight important parameters such as required length of protection for varying SMC schedules, and show that multi-stage therapeutics can increase the effectiveness of SMC.

Notably, results describe required profiles of such therapeutics to be most effective in combination with SMC, and that if SMC reaches high coverage and adherence to all cycles, the added benefit will be marginal. This is relevant for considerations on assessing cost-effectiveness of a new therapeutic, which will depend on the public health effectiveness of existing interventions (such as SMC).

Overall the manuscript is very well written, clearly describing the methods and results. The figures effectively show the results from the exhaustive parameter explorations, providing many important insights on the most relevant parameters, and parameter thresholds. The figure captions are very clear and helpfully include notes for correct interpretation of metrics a reader might be less familiar with (i.e. of the Sobol effect).

The authors also address important limitations in the discussion, such as SP-AQ drug resistance, waning immunity of the vaccine booster, or transmission blocking therapeutics. Of those, it would have been most interesting to see scenarios under varying SP-AQ resistance profiles. The authors provide a hypothesis in the discussion section (L414-416), but this could also have been backed up by simulations, or elaborated on further.

Thank you very much for your constructive feedback. We are very grateful for your careful and thoughtful comments, which we have addressed in detailed below.

We particularly appreciate your suggestion to elaborate on the potential role of resistance to SP-AQ, and have added a paragraph to our discussion to clarify how our results relate to settings where SP's protection is shortened by resistance. In our new paragraph (lines 435-442), we explain why the added value of a novel therapeutic is likely to be greatest in these settings, where children experience longer unprotected intervals between SMC rounds:

> *"Although we did not explicitly model resistance to SP-AQ, our results suggest that a novel therapeutic would have its strongest benefits in regions where SP's duration of protection is shortened by resistance. In such settings, children experience longer periods of vulnerability between SMC rounds, increasing both the absolute and relative benefit of an additional intervention. Reduced SP efficacy would lower the impact of SMC alone, meaning that comparable reductions in malaria burden could be achieved with a novel therapeutic that has a shorter protection half-life or lower efficacy than would be required in fully SP-sensitive settings. As the clinical pipeline of next-generation vaccines and long-acting injectables matures, explicitly evaluating these interactions with varying levels of resistance to SP will be an important area for future modelling."*

How does transmission intensity influence the results? Figure 4.4, includes variation by transmission intensity, but authors do not pick up on this in the discussion. It seems to be worth mentioning at least that the trends remain mostly constant across transmission settings/ does that hold if the treatment coverage were higher or seasonality longer? Interestingly, for cumulative severe cases 10 years old, blood stage therapeutics effects compared to SMC increases by transmission intensity, but not in 5 year olds.

Prof Melissa Penny
Fiona Stanley Chair in Child Health Research
UWA Centre for Child Health Research
melissa.penny@uwa.edu.au

Thank you for this helpful observation. We agree that some important dynamics vary across transmission intensity and that we had not sufficiently highlighted these in our original text. We have now integrated these points more clearly into both the results and discussion. Specifically, we added two paragraphs to the results section on 'Combining SMC with seasonal deployment of a multi-stage therapeutic' (lines 354-361 and 385-388), describing how the relative influence of pre-liver and blood-stage properties changes across transmission settings. We also added a short statement to the discussion (lines 431-434) to emphasise the importance of these patterns. The revised discussion text now reads:

> *"In addition, the increased benefit of a blood stage or combination therapeutic for benefit on severe disease outcomes at ten years old may also reflect the role of blood stage activity in preventing high-density infections once an SMC program has ended and children are again exposed to frequent infections in high transmission environments."*

Relatedly, how do seasonality and health system scenarios influence the results? I.e. Figure 4.2 would be interesting to see with additional bars (i.e. not filled dots) for case management or seasonality. Figure 2.7 does show both levels for seasonality and also for health system scenarios, and would benefit from additional discussion. It is plausible that with imperfect deployment, the added benefit of therapeutics is larger if seasonality is longer, but this shows only if access to care is high, not low. It would be useful if the authors could elaborate on this in the discussion.

Thank you for highlighting this. We agree that seasonality and access to care can influence the magnitude of impact, and we now clarify this in the results (lines 313-315), where we note that the general trends remained consistent across these settings, with somewhat larger effects in longer seasons and under higher access to care. We appreciate the suggestion to further expand on these dynamics in the discussion. However, given the number of parameters already explored in the main text, the complexity of the multi-panel figures and the length of the discussion, we felt that adding additional layers of interpretation there would reduce clarity. For this reason, we have chosen to keep the detail of these results in the supplement (Figures 2.7 and 2.8) and to maintain a simpler, higher-level message in the discussion.

The supplementary figure 2.8 shows the predicted relationships between pre-liver stage protection half-life, initial efficacy and reductions in cumulative severe cases by ten years old, compared across SMC deployment scenarios for a product with a rapid decay in protection. The figure suggests a slight disadvantage (-5) compared to SMC alone? This is not commented on in the discussion. As it is clear from the discussion, that long acting therapeutics are to be prioritized for combination with SMC, the importance of avoiding rapid decay, even if half life is long, could benefit from further elaboration in the discussion (i.e. L420) and reference in the main text. As it is now, the decay shape is not discussed in the discussion section.

Thank you very much for this observation. The shape of decay does indeed play an important role for sustaining impact beyond the period of intervention deployment. In our analyses, both pre-liver and blood stage therapeutics required a long tail of protection to maintain reductions in cumulative burden as children aged. We describe this dynamic in detail in our manuscript's results section at lines 281-290, and discuss its implications for product development in our discussion in lines 394-397 and 401-406.

Looking forward, authors cite previous work they have published on next generation SMC scenarios, could the authors comment how results may change when considering next generation SMCs, or mention this as an area of further research and consideration given that next generation SMCs are also in development.

Thank you for this thoughtful suggestion. We have updated the relevant paragraph in our discussion to make the link to next-generation SMC products explicit (lines 449-451), where we now recognise that improved SMC regimens might change the relative added value of a new therapeutic.

In the methods high transmission intensity is described as 64%, and not 32% which is also labelled high. Perhaps in the methods 64% could be described as very high, for distinction of those intensity levels? Relatedly, in Fig 4.4, prevalence levels include 33, instead of 32 mentioned in the main text, and is only starting at 15%, not 9% which is used in the methods.

THE UNIVERSITY OF WESTERN AUSTRALIA

Prof Melissa Penny
Fiona Stanley Chair in Child Health Research
UWA Centre for Child Health Research
melissa.penny@uwa.edu.au

Thank you for noting these inconsistencies. The transmission intensities shown in the figures reflect the prevalence that was realised within each model scenario, rather than fixed input values. Because the individual-based model includes stochastic variation, and because transmission depends on both access to care and the seasonality profile, the resulting *Pf*$PR_{2\text{-}10}$ values vary slightly across scenarios. For this reason, the transmission values shown in the results (e.g., 33% instead of 32%, or 15% instead of 9%) represent the model-generated outcomes rather than exact preset inputs.

To avoid confusion, we have removed the specific mention of percentage values for transmission intensity scenarios in the methods (lines 216, 217) and have added a sentence to clarify this point in lines 219, 220:

> *"Since PfPR$_{2\text{-}10}$ is calculated from simulated outcomes, it varies with both model scenario and stochasticity." Where relevant, we have also revised our use of the labels "high" and "very high" transmission for consistency.*

Lines 32-33 (now 31-33): the authors write that "highlighting the need to prioritise the clinical development of these therapeutics for combination with malaria chemoprevention", or should it rather be that "clinical development should prioritise agents with demonstrated suitability for combination with malaria chemoprevention". Whether to prioritise clinical development, or whether to prioritize specific therapeutics during clinical development is a slightly different nuance.

Thank you for this suggestion. We have now rephrased the final sentence of our abstract to read as follows:

> *"These results indicate that combining SMC with malaria therapeutics active against multiple stages of the parasite life cycle can improve the effectiveness of SMC, and highlight the need for clinical development to prioritise multi-stage therapeutics for improved malaria prevention in children."*

Line 54 (now 54, 55): could consider updating to estimates from latest World Malaria Report

Thank you for catching this, we have updated this line to refer to the 2025 World Malaria Report.

Lines 150-153 (now 150, 152): To what do 1) and 9) refer to?

These numbers refer to the decay shape parameter for the two exemplar therapeutic profiles we reference in this paragraph; the brackets are the closing brackets for the specific parameters that define these profiles. We understand this may have been confusing and so have written the numbers out as the word 'one' and 'nine'.

Line 210: how many stochastic replicates were run? - not mentioned in the main text, but in the supplement.

Line 210 now specifies that ten stochastic replicates were run.

Line 220 (now 221): the text mentioned 4 seasonal profiles which appears to be a typo and it should be 2

Thank you for bringing this to our attention. We have removed the word "seasonal" in this paragraph to make it clear that this line refers to the four SMC deployment scenarios.

Line 141 (now 140-142): to what do the explicit models of therapeutic activity refer to ? to the 500 parameters samples?

Thank you, we agree that our phrasing in this sentence was unclear. It now reads:

> *"To model novel malaria prevention therapeutics, we simulated a range of malaria vaccine, monoclonal antibody, and long-acting injectable drug candidates by combining modelling their activity against different stages of the Plasmodium falciparum parasite life cycle."*

Figure 2B: are the illustrative cumulative cases based on a simulation with transmission intensity corresponding to PfPR2-10 32%?

Prof Melissa Penny
Fiona Stanley Chair in Child Health Research
UWA Centre for Child Health Research
melissa.penny@uwa.edu.au

Yes, this is correct. We have now adapted lines 278, 279 in our figure caption to read "Model results are shown for a scenario with transmission intensity corresponding to 32% *Pf*$PR_{2\text{-}10}$". We have also edited the correspond figure captions in our supplement in the same way.

The random allocation does seem to be missing for Predicted cumulative case curves by age in the Supplement.

Thank you for noting this, the omission was intentional. The cumulative case curves in our supplementary figures 2.1 and 2.2 track outcomes for a defined cohort of children over time. Producing these curves requires individual-level infection histories. In the random allocation scenario, however, SMC is assigned probabilistically each year (to between 30% and 100% of children) and OpenMalaria does not output individual-level data – only aggregated results by user-defined age groups and time intervals. Because we cannot identify which specific children did or did not receive SMC in this scenario, it is not possible to reconstruct cumulative case trajectories for a consistent cohort. For this reason, we did not include cumulative case curves for the random allocation scenario. All other analyses are shown for all four SMC deployment scenarios.

Prof Melissa Penny
Fiona Stanley Chair in Child Health Research
UWA Centre for Child Health Research
melissa.penny@uwa.edu.au

Reviewer #3

This study sought to provide evidence on the potential benefit of combining novel malaria therapeutics with SMC activities. An individual based transmission model was used to estimate the public health impact of combining SMC with new therapeutics to reduce uncomplicated and severe cases during and five years after deployment. Therapeutics targeting pre-liver and/or blood stages were characterised based on initial efficacy, duration of efficacy, and protection half-life to identify suitable candidates required to achieve target reductions in infections among children under 10. The model simulates a cohort of children born at the beginning of intervention to measure reductions in uncomplicated and severe cases under four deployment scenarios. Three key observations were made in the study, firstly, a therapeutic with >50% initial efficacy and >230 days protection half-life led to >23% reduction in severe cases during, and >5% five years after intervention stopped in a high transmission setting with three cycles of SMC. Secondly, therapeutics with longer protection provide additional benefit in regions with four or five SMC cycles. And thirdly, multi-stage therapeutics showed substantial public health impact, particularly in regions where SMC efficacy is compromised. As a result, it is concluded that therapeutics targeting both pre-liver and blood stages need to be prioritised for development and selected for combination with SMC.

The study uses a mathematical model to address the challenge of adherence to SMC based on data from an RCT for combining SMC with a vaccine, phase II trial on monoclonal antibodies, and the relative effectiveness of long-acting injectables.

Utilising an established model such as OpenMalaria, which has been validated on historical data added credibility to model outcomes. Furthermore, calibrating vaccine booster efficacy and properties to clinical trial data provided a reasonable baseline for impact.

Simulation results explored the impact of pre-liver stage, blood stage, and multistage therapeutics, which enabled a comprehensive analysis into the potential benefits of combining SMC with novel therapeutics.

Therefore, I strongly recommend that this manuscript be accepted for publication.

Thank you very much for your positive feedback. We are grateful for your time and thoughtful comments, which we have addressed in detailed below.

Lines 28, 29: should rather be rewritten as "... to reduce cumulative severe cases by >23% during interventions, and >5% five years after deployment stopped.", for clarity.

Thank you, we have adopted this phrasing.

Lines 304-311 (now 303-310): do not use the terms used in Figure 4. This makes it difficult for the reader to follow without referring to the caption of Figure 1 (lines 201 – 205), where the scenarios are clearly defined in terms of the SMC cycles, coverage, etc. How the results are currently presented does not seem to link with Figure 4, so the authors should rephrasing these lines for clarity and provide a direct link with the results presented in the image.

We agree that our manuscript was not clear here and have rephrased the text in this paragraph to connect it with the scenario labels used in Figure 4. Lines 305, 306 now include "… deployed as in our imperfect SMC deployment scenario, with three yearly cycles of SMC…" and lines 308, 309 include "When the same therapeutic was combined with five SMC cycles, as in our perfect SMC deployment scenario…"

Lines 345-347 (now 348-353): The attributed percentage is 95% for uncomplicated cases, and the 70% mentioned in lines 345 – 347 is for severe cases. Also, this is particularly measured at five years old, so the text should reflect that.

Thank you for highlighting this, we have rephrased this text to clarify which percentage is attributed to uncomplicated vs. severe disease, and to outcomes measured in children five vs. ten years old. Lines 348-353 now read as follows:

Prof Melissa Penny
Fiona Stanley Chair in Child Health Research
UWA Centre for Child Health Research
melissa.penny@uwa.edu.au

> *"More than 88% of variation in uncomplicated cases measured by five years old was attributed to the combined impact of the pre-liver stage component's initial efficacy and protection half-life (Fig 6, panel A), and more than 75% of variation in cumulative uncomplicated cases measured by ten years old. However, blood stage activity was crucial for impact on cumulative severe cases by the time children were ten years old; up to 89% of variation in the reduction in cumulative severe cases at ten years could be attributed to the blood stage component's initial efficacy and protection half-life (Fig 6, panel A)."*

The authors should consider mentioning the baseline annual prevalence as the variations at ten years old differ distinctly at 15% and 64% annual prevalence for severe cases.

We agree that this dynamic should be described in our manuscript, and another reviewer also highlighted the interesting dynamics that emerge across levels of transmission intensity. To better integrate these dynamics into both our results and discussion, we have included two additional paragraphs in our results section on 'Combining SMC with seasonal deployment of a multi-stage therapeutic', in lines 354-361 and 385-388. We have also pointed to the importance of these results in lines 431-434 in our discussion, which now read:

> *"In addition, the increased benefit of a blood stage or combination therapeutic for benefit on severe disease outcomes at ten years old may also reflect the role of blood stage activity in preventing high-density infections once an SMC program has ended and children are again exposed to frequent infections in high transmission environments."*

Introduction: The lack of adherence to SMC and the threat of parasite resistance to SP-AQ have prompted a need for novel therapeutics to be used in combination with SMC.

A Randomised Control Trial (RCT) was conducted to evaluate the benefits of combining SMC with a vaccine. SP-AQ clears blood stage and prevents pre-liver emergence, while the RTS,S vaccine prevents liver stage infection. This combination was found to be more effective than either intervention alone. However, it is not yet known whether similar benefits can be observed for other therapeutics.

The first phase II results from pre-liver stage monoclonal antibodies are available. These show seasonal suitability with high levels of protection following single dose, but cost-effectiveness concerns remain.

Atovaquone-proguanil injectables offer three to four months of protection at lower cost than monoclonal antibodies and target multiple stages.

These three therapeutics act differently at pre-liver and blood stages, and how they can improve the benefits of SMC is important to know, so an individual based transmission model is used to address this.

Thank you very much for this helpful summary.

Materials & methods: OpenMalaria is an established model with a detailed framework that has been validated on historical data. It has multiple components to cater for different stages of the parasite lifecycle. Hence, it was appropriate for this research study.

Although immunity is part of the OpenMalaria framework, it is not modelled in the study. The authors have acknowledged this as a limitation of their research, and future studies are urged to validate changes in model outcomes when immunity is accounted for.

The activity of each therapeutic at various stages is modelled independently, so any synergistic effects emerge as a result of the OpenMalaria model structure. LHS is a statistical sampling method used to estimate parameter values and a table of sampled parameter ranges is provided for reproducibility.

Thank you very much for your summary; we agree with your comments.

THE UNIVERSITY OF WESTERN AUSTRALIA

Prof Melissa Penny
Fiona Stanley Chair in Child Health Research
UWA Centre for Child Health Research
melissa.penny@uwa.edu.au

Bayesian optimisation was used to calibrate vaccine booster efficacy and properties to data from a clinical trial. The authors claim that this process was able to reproduce the observed monthly incidence despite not providing evidence of this.

Thank you for raising this. We have summarised our model validation in the section titled ‘Intervention model validation’ (lines 172-188), where we describe how a Bayesian optimisation procedure was used to calibrate vaccine efficacy and duration to the RTS,S/AS01 seasonal vaccination trial, and how the calibrated model reproduced the observed monthly incidence across trial arms. These results were published in detail in Malinga et al. (2025), using the same individual-based model as we have used for this study. The methods for the validation component of this research are included in Malinga and colleagues’ supplement. For reference, we have included Figure 2 from this paper below. The points in panel A show the observed monthly incidence from the trial, and the lines show our model’s fit to this data. The close alignment between our simulated incidence and that observed in the trial indicate good model fit.

Reference: Malinga J, Braunack-Mayer L, Masserey T, et al. PLOS Global Public Health. 2025. https://doi.org/10.1371/journal.pgph.0004549

Fig 2 from Malinga et al. (2025). Monthly clinical incidence and hazard ratios for the clinical trial data (black dots) compared with model simulations (blue lines/triangles) using the best fit assumption for the efficacy against infection of annual booster doses four and five and SPAQ preventive half-life duration.

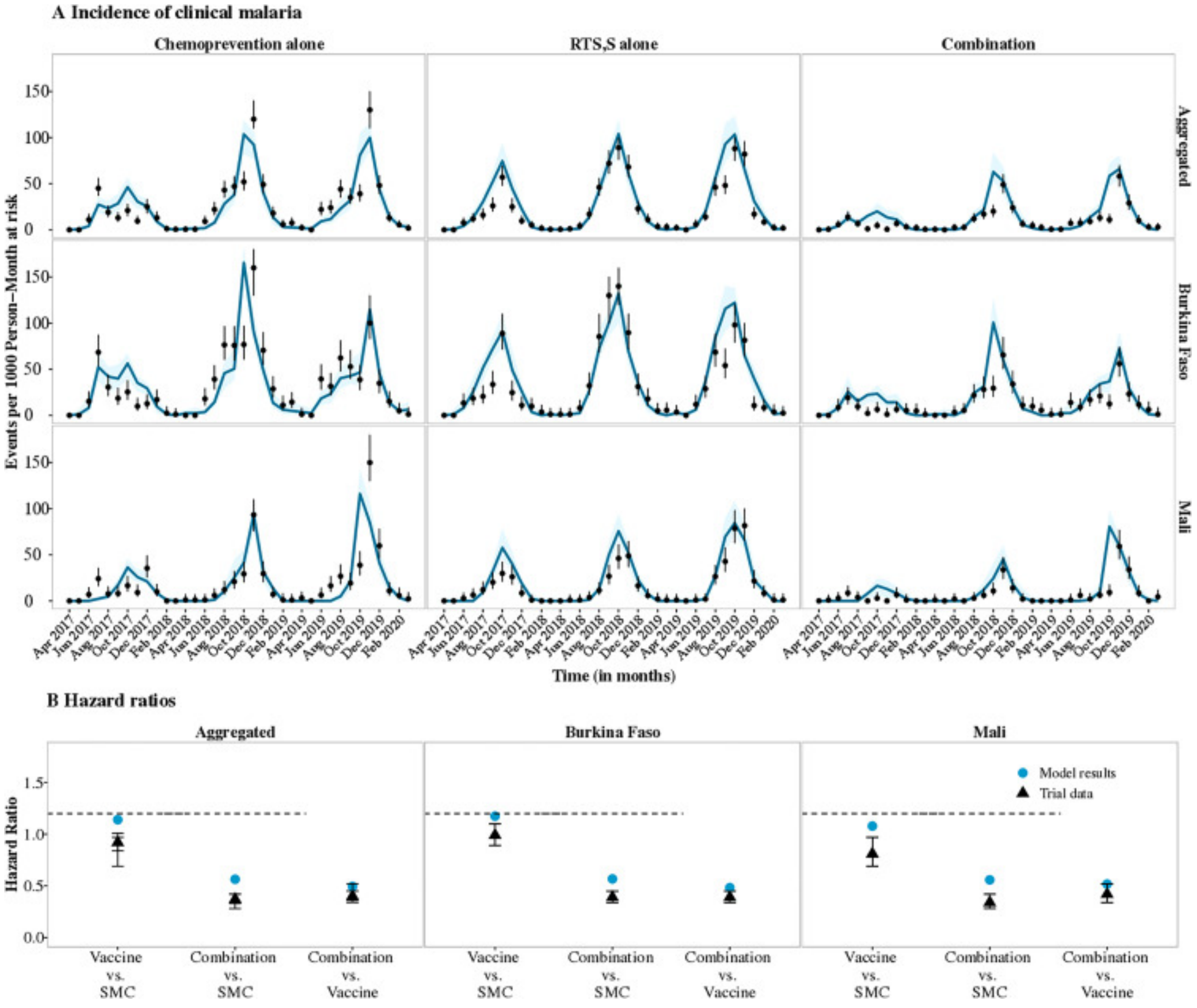

Prof Melissa Penny
Fiona Stanley Chair in Child Health Research
UWA Centre for Child Health Research
melissa.penny@uwa.edu.au

A) Best fit parameters for Burkina Faso (high prevalence and seasonal transmission), Mali (moderate prevalence and highly seasonal transmission) and both countries aggregated shown for the three trial arms, B) Hazard ratios between the trial arms for both countries separately and combined. The black dots shown with 95% confidence intervals represent the trial field data and the blue lines/triangles illustrate the modelled output from the simulations with the shaded region showing the confidence intervals averaged over 100 seeds. In this figure, the parameters were optimized for the arms where seasonal chemoprevention or vaccination were given alone and used to simulate the model results for the arm where the vaccine and chemoprevention were combined.

Results: Figure 2 shows the outcomes of combining SMC with a pre-liver stage therapeutic. Two therapeutics were considered, and reductions were observed by age five when the intervention stops. Long duration, long tails, and high efficacy were crucial to maintaining reductions in infections. Also, higher efficacy could be preferred for shorter durations to achieve the same long-term benefits 5 years after intervention has stopped. However, longer duration is required to benefit in settings with high SMC coverage.

Figure 5 shows the outcomes of combining SMC with a seasonal blood stage therapeutic Blood stage therapeutics primarily serve to supress disease severity rather than prevent infections. High initial efficacy is required to impact infections in the long-term but reductions in severe cases could be achieved with lower efficacy.

Figure 6 shows the outcomes of combining SMC with a multistage therapeutic. Pre-liver activity of multistage therapeutics is attributed to substantial reductions in infections during intervention. While long term reductions in severe cases are attributed to blood stage activity after intervention has stopped. It is concluded that multistage therapeutics provide greater impact than single stage ones, particularly pre-liver stages the benefits of which are greatly improved.

Thank you for your helpful summary.

Discussion: Pre-liver stage vaccines are proposed as candidates to partner with SMC to reduce uncomplicated cases. Extended pre-liver or multistage activity is required to sustain benefits throughout childhood. Multistage therapeutics are further proposed for selection over single stage therapeutics. Integrating immunity into the model would have implications for outcomes related to multistage therapeutics because blood stage activity provides protections against parasites that escape pre-liver stage protection. Due to the paucity of data on clinical trials as mentioned in the discussion, the authors suggest that the development of multistage therapeutics, and more specifically, blood stage active long-acting injectables could fill this gap.

Layering SMC with a vaccine or monoclonal antibodies would benefit the greatest in regions where SMC coverage is compromised, either due to lack of adherence or parasite resistance. In regions where coverage is high, then long-tailed therapeutics are needed to benefit from combinations with SMC. Given the cost implications of four or five SMC cycles, these trade-offs beg the question of the cost-effectiveness of combining therapeutics with SMC at different coverage levels. However, this was not modelled in the study due to uncertainties related to the costs of developing new therapeutics.

Since this study focused on a high transmission areas and children under 10 years old, the authors suggest that future modelling studies should explore these combination strategies in perennial settings and target specific age groups. Multistage therapeutics are recommended for combination with SMC to maintain benefits in children, 5 years after intervention has stopped.

Thank you once again for this summary, which we agree with.
